# Regenerative Activities of ROS-Modulating Trace Metals in Subcutaneously Implanted Biodegradable Cryogel

**DOI:** 10.3390/gels8020118

**Published:** 2022-02-14

**Authors:** Abdulla A. Yergeshov, Mohamed Zoughaib, Rezeda A. Ishkaeva, Irina N. Savina, Timur I. Abdullin

**Affiliations:** 1 Institute of Fundamental Medicine and Biology, Kazan (Volga Region) Federal University, 18 Kremlyovskaya St., 420008 Kazan, Russia; abdulla.ergeshov@mail.ru (A.A.Y.); zmokhamed@kpfu.ru (M.Z.); rezaahmadishina@kpfu.ru (R.A.I.); 2 School of Applied Sciences, University of Brighton, Huxley Building, Lewes Road, Brighton BN2 4GJ, UK; i.n.savina@brighton.ac.uk

**Keywords:** trace metals, cryogels, reactive oxygen species, tissue regeneration, skin, angiogenesis, immune cells

## Abstract

Divalent trace metals (TM), especially copper (Cu), cobalt (Co) and zinc (Zn), are recognized as essential microelements for tissue homeostasis and regeneration. To achieve a balance between therapeutic activity and safety of administered TMs, effective gel formulations of TMs with elucidated regenerative mechanisms are required. We studied in vitro and in vivo effects of biodegradable macroporous cryogels doped with Cu, Co or Zn in a controllable manner. The extracellular ROS generation by metal dopants was assessed and compared with the intracellular effect of soluble TMs. The stimulating ability of TMs in the cryogels for cell proliferation, differentiation and cytokine/growth factor biosynthesis was characterized using HSF and HUVEC primary human cells. Multiple responses of host tissues to the TM-doped cryogels upon subcutaneous implantation were characterized taking into account the rate of biodegradation, production of HIF-1α/matrix metalloproteinases and the appearance of immune cells. Cu and Zn dopants did not disturb the intact skin organization while inducing specific stimulating effects on different skin structures, including vasculature, whereas Co dopant caused a significant reorganization of skin layers, the appearance of multinucleated giant cells, along with intense angiogenesis in the dermis. The results specify and compare the prooxidant and regenerative potential of Cu, Co and Zn-doped biodegradable cryogels and are of particular interest for the development of advanced bioinductive hydrogel materials for controlling angiogenesis and soft tissue growth.

## 1. Introduction

Treatment of severe organ/tissue injuries generally requires the replacement of a post-traumatic defect with a scaffold such as an autologous/decellularized graft or preferably biomimetic biodegradable material capable of supporting cell growth and functional activity while overcoming the limitations of donor grafts mostly related to their scarcity and host immunogenic responses [1]. Although a number of biomimetic scaffolds composed of synthetic or/and naturally occurring biopolymers have been proposed, these materials by themselves are not able to provide sufficient regenerative responses without special bioactivation [2].

Transplanted cells such as mesenchymal stem cells from different sources [3], skin fibroblasts [4], neural cells [5], as well as related products (e.g., platelets [6] and extracellular vesicles [7]), were proved to enhance tissue regeneration activity of biomaterials. However, these biological products unavoidably feature typical limitations of donor tissues, primarily, low availability, variability of characteristics, and health risks. Given that the regenerative potential of the transplanted cells is mainly attributed to secreted signaling molecules, recombinant growth factors can be used instead to improve tissue-replacing scaffolds [8], although this is complicated by the increased manufacturing cost of pure growth factors and their deactivation upon immobilization and storage. Therefore, more stable, reproducible and available active components of biomaterials are still demanded in tissue engineering and regeneration applications.

Trace metals (TM) are essential bioactive microelements involved in the maintenance and regulation of cell metabolism, the functioning of the immune system, turnover and the regeneration of soft and hard tissues. Since TM deficiency accompanies many degenerative and traumatic diseases, their local administration in combination with biomaterials represents a promising therapeutic strategy [9,10]. The regenerative activities of such TMs (primarily, divalent ions of Co, Cu, Zn, Mn, and Fe metals) were established mainly for solid osteoinductive materials based on inorganic scaffolds doped with metal ions or nanoparticles. For instance, collagen scaffolds functionalized with Cu-eluting bioactive glass particles possessed profound in vitro angiogenic activity toward rMSCs and HUVECs as well as antibacterial and osteogenic effects in vivo [11]. Implanted Co-containing borosilicate glass-based scaffolds remarkably enhanced bone regeneration and the vascularized network of the calvarial defective site in rats [12]. Likewise, Zn incorporation into Ca-silicate-based cements increased the osteostimulative activity of the composite material in a maxillofacial bone defect model in rabbits [13].

Hydrogels have been considered among the most promising materials for tissue repair, showing successful results in pre-clinical trials owing to their appropriate physicochemical and hydration properties similar to those of soft body tissues [14,15]. Earlier, gelatin methacrylate hydrogels embedded with Cu nanoparticles supported the attachment and proliferation of 3T3 fibroblasts and inhibited bacterial growth in vitro, in addition to promoting effective wound closure in mice without inflammatory response [16]. Zn cross-linked alginate-polyacrylamide hydrogel supported increased vascular growth, collagen deposition, granulation tissue formation and wound healing along with reduced inflammation [17]. The co-encapsulation of Co and Ca ions within gauze-alginate composite hydrogel resulted in enhanced local VEGF and TGF-β1 protein expression and accelerated wound healing in a mouse bacteria-infected wound model [18]. We have shown recently that macroporous hydrogels prepared by the cryogelation technique, namely, cryogels composed both of bio- and synthetic polymers, represent a promising type of scaffold for bulk bioactivation with TMs in a controllable and stable manner [19,20]. The advanced porous structure of cryogels ensures enhanced mammalian cell infiltration and activity of the incorporated metal dopant within the scaffold [19,20,21]. The increased healing of an excisional skin defect treated with the Zn-doped gelatin cryogel [19] as well as enhanced in vitro angiogenic responses of poly(2-hydroxyethyl methacrylate) cryogels modified with Cu^2+^ (via complexation with GHK peptide) [20] were demonstrated.

Further clarification of mechanisms of local regenerative and adverse effects of TM-containing hydrogels is demanded. These effects are often controversial, depending on biomaterial formulations, which may, for instance, show both prooxidant [22,23,24] and antioxidant [25,26] activities for the same TMs. Our previous studies prove cryogels as a relevant platform both for the examination of therapeutic effects of the TM compounds and for the development of advanced bioinductive materials [19,20].

In this work, we conducted a comparative investigation of regenerative activities of biodegradable gelatin cryogel doped with Zn, Cu or Co divalent metals as one of the most therapeutically relevant TMs [10]. Considering that TMs can participate in non-enzymatic redox-reactions, such as the Fenton-type generation of reactive oxygen species (ROS) involved in cell signaling [27,28,29], the TM-doped cryogels were assessed in relation to redox-modulating and cytokine-regulating in vitro activities of the metal dopants. To charactrize in vivo regenerative activities of the TM-doped cryogels, a subcutaneous implantation model with a comprehensive histological evaluation was optimized, considering the relevance of this model for understanding fundamental effects of biomaterials on host tissue responses related to cellularization, angiogenesis, and inflammation [30,31,32]. Thus, specific localized effects of the cryogel-formulated TMs on different skin structures and underlying tissues were studied and compared.

## 2. Materials and Methods

### 2.1. Materials

Bovine skin gelatin, 3-(4,5-dimethylthiazol-2-yl)-2,5-diphenyl tetrazolium bromide (MTT reagent), 4′,6-diamidino-2-phenylindole (DAPI), phenazine methosulfate (PMS), and Triton X-100 and 2′,7′-dichlorofluorescin diacetate (DCFDA) were purchased from Sigma-Aldrich. Monochlorobimane (MCB) was purchased from ThermoFisher Scientific. CuSO_4_·5H_2_O, ZnCl_2_, CoCl_2_·6H_2_O, glutaric dialdehyde (GDA), and cresyl violet acetate were obtained from Acros Organics. Citrus pectin (classic CM 201) was obtained from Herbstreith&Fox.

3-(4,5-Dimethylthiazol-2-yl)-5-(3-carboxymethoxyphenyl)-2-(4-sulfophenyl)-2H-tetrazolium (MTS reagent) was purchased from Promega. Phalloidin CruzFluor™ 647 conjugate, anti-VEGF (C-1) mouse monoclonal, anti-ICAM-2 (S-16) goat polyclonal, anti-MMP-3 goat monoclonal and anti MMP-3 goat monoclonal antibodies were purchased from Santa Cruz Biotechnology. Anti-HIF-1a mouse monoclonal antibody, donkey anti-mouse IgG (H + L) highly cross-adsorbed secondary antibody, Alexa Fluor 647, and donkey anti-goat IgG (H + L) cross-adsorbed secondary antibody, Alexa Fluor 555, were obtained from ThermoFisher Scientific. Anti-CD31 (PECAM-1) rabbit monoclonal antibody was obtained from Abcam. Hematoxylin and Eosin, and Giemsa staining were purchased from BioVitrum (Russia). Cell culture media and reagents were purchased from Paneco (Russia).

### 2.2. Preparation and Characterization of Cryogels

Cryogels were prepared from bovine skin gelatin using cryotropic gelation method as previously described [19] with some modifications. Briefly, the reaction mixture contained gelatin (2.5 wt%), pectin (ca. 0.1 wt%) and TM (0.04–1 mM) in aqueous solution. The gelation was initiated by adding 0.25 wt% GDA to the solution upon stirring followed by its pouring into a glass Petri dish and cooling at a temperature of −12 °C for 4 h in a thermostat and then at −18 °C for additional 24 h in a freezer. The resultant ~3 mm thick cryogel sheet was thawed at room temperature, washed and stored in 25% ethanol solution in the fridge.

Rheological properties of the cryogels were analyzed using MCR 302 rotational rheometer (Anton Paar) at 25 °C. The strain sweep and frequency sweep tests were performed by applying 0.01–100% strain amplitude (ω = 10 rad s^−1^) and 0.01–100 rad s^−1^ angular frequencies (γ = 1%), respectively. The storage (G′) and loss (G′′) modulus of the materials were presented as a function of strain and frequency. Frequency dependences of G′ and G′′ were detected within linear viscoelastic region (LVR). Porous structure of the cryogels was analyzed using laser scanning confocal microscopy (LSCM) using LSM 780 microscope (Carl Zeiss) equipped with argon laser excitation (488 nm). Zeiss ZEN black software was used for acquisition. Pore size of the cryogels was evaluated using ImageJ software (NIH, USA).

### 2.3. Cell Maintenance and Seeding

NIH 3T3 mouse embryonic fibroblasts (ATCC) and primary human skin fibroblasts (HSFs) isolated as described earlier [33] were grown in α-MEM supplemented with 10% FBS, penicillin (100 U/mL)/streptomycin (100 µg/mL) and L-glutamine (2 mM). Freshly isolated human umbilical vein endothelial cells (HUVECs) were kindly provided by Dr. Ilnur Salafutdinov (Kazan Federal University). HUVECs were grown in RPMI 1640 supplemented with 20% FBS, penicillin (100 U/mL)/streptomycin (100 µg/mL), L-glutamine (2 mM), sodium pyruvate (2 mM), heparin (100 µg/mL), and 30 µg/mL endothelial cell growth supplements (ECGS). The cells were cultured in a temperature- and humidity-controlled incubator at 37 °C. The culture medium was refreshed every 2 days. Primary cells (HSFs and HUVECs) were studied between passages 3 and 6.

Prior to cell seeding, round cryogel sheets (14 mm in diameter) were incubated in penicillin (2.5 kU/mL)/streptomycin (2.5 mg/mL) antibiotic mixture for 1 h, rinsed with HBSS and equilibrated in the culture medium. Cells were seeded onto the cryogel surface using top seeding method in 24-well plate at a density of 4.88 × 10^4^ cells/cm^2^ of cryogel area and incubated for 1.5 h under standard culture conditions to allow for cell attachment.

### 2.4. Cell Detection in Cryogels

The cryogels with cultured cells were collected at day 3, transferred into new wells containing 0.5 mL MTS/PMS reagents in fresh culture medium to assess cell metabolic activity [34]. After incubation for 1.5 h under standard culture conditions (37 °C, 5% CO_2_), the absorbance was determined at 490 nm on an Infinite M200 PRO microplate analyzer (Tecan).

For bright-field microscopy analysis, the cryogels with cultured HUVECs were fixed with 4% *p*-formaldehyde for 2.5 h and gently washed with PBS. The fixed cells were subsequently stained with cresyl violet (0.1% *w*/*v* in ultrapure water) for 5 min and visualized using AxioObserver Z1 microscope (Carl Zeiss).

### 2.5. Immunocytochemistry

The fixed cryogel matrices were incubated in 0.1% Triton X-100 in PBS for 15 min for cell membrane permeabilization and washed three times with PBS. Non-specific binding sites in the materials were blocked using 1.5% bovine serum albumin (BSA) for 30 min at room temperature. The matrices were subsequently incubated with primary antibodies (1:500 in 1.5% BSA/PBS) against VEGF or ICAM-2 overnight at 4 °C followed by incubation with Alexa Fluor 647-conjugated donkey anti-mouse or Alexa Fluor 555-conjugated donkey anti-goat secondary antibodies (1:350 in 1.5% BSA/PBS) for 45 min at room temperature. Following washing with PBS, the cell nuclei were stained with 4′,6-diamidino-2-phenylindole (DAPI). For cytoskeleton visualization, F-actin was labeled using phalloidin CruzFluor™ 647 conjugate in 1% BSA for 30 min. LSCM images were acquired on an LSM 780 microscope.

### 2.6. Detection of ROS and Glutathione

To assess extracellular ROS-generating ability of the TM-doped cryogels, the materials in a 24-well plate were incubated with H_2_O_2_ (21.5 mM) in PBS for 60 min in the presence of 5 µM DCFDA. The probe fluorescence (λ_ex_/λ_em_ = 490/526) in the solution was monitored during the reaction using an Infinite M200 PRO microplate analyzer.

Intracellular effects of dissolved TMs on both ROS and reduced glutathione levels were additionally studied. 3T3 cells were seeded in 96-well plate at a density of 2 × 10^4^ cells per well and grown overnight. The cells were exposed to dissolved CuSO_4_, ZnCl_2_ or CoCl_2_ at a concentration of 1 or 10 μM in HBSS for 60 min in CO_2_ incubator. Subsequently, the treated cells were stained with 20 µM DCFDA fluorescent probe for 40 min or with 5 µM monochlorobimane probe for 60 min to assess intracellular ROS and reduced glutathione, respectively. The cellular fluorescence of DCFDA (λ_ex_/λ_em_ = 490/526) and MCB (λ_ex_/λ_em_ = 380/480) was detected. The data are presented as mean ± SD.

### 2.7. Multiplexed Fluorescent Bead-Based Immunoassay

Top seeded HSFs were grown within non-doped and metal-doped cryogels as mentioned above in 2.3. At 24 h post-seeding, the conditioned culture medium containing cell secretion was collected and immediately frozen at –80 °C. The analysis of secreted levels of cytokines was performed using xMAP Luminex technology on a Bio-Plex MAGPIX analyzer (BioRad, USA) according to the manufacturer’s recommendations. A commercially available MILLIPLEX MAP Human Cytokine/Chemokine Magnetic Bead Panel (HCYTMAG-60K-PX41) was used to quantitatively measure cytokine/chemokine levels. Standard reference curve was used to determine the concentration of analytes in each sample according to their fluorescence intensities. Background levels of analytes in cell-free culture medium were subtracted. Bio-Plex Manager 4.1 software (Bio-Rad Laboratories) was used to analyze the data.

### 2.8. In Vivo Study

#### 2.8.1. Animals

Wistar male rats (340 ± 38 g) were purchased from Vivarium of Academy of Medical and Technical Sciences (Russia). Animal care was performed according to European regulations on the protection of experimental animals (Directive 2010/63/UE) and Russian regulations (No. 742 from 13.11.1984, Ministry of Education and Science). The rats were divided into four groups and were kept in plastic cages under controlled conditions (at a temperature of 20 ± 3 °C and a humidity of 65 ± 10%) with running water and complete feed. The in vivo study was approved by the Institutional Ethical Review Board of the Kazan Federal University.

#### 2.8.2. Subcutaneous Implantation Model

The animals were anesthetized using tiletamine-zolazepam-xylazine (30/20/10 mg/kg, respectively) administered via IP injection. The upper back skin was shaved and disinfected with 70% ethanol solution. Two symmetrical full-thickness skin incisions were made horizontally with a width of 1.5 cm using fine scalpel. A subcutaneous pocket with about 1.5 cm long from the lower incision border was created on each side by detaching the skin from the underlaying tissues employing anatomical forceps (Figure 7). The incision was disinfected with 0.05% chlorhexidine solution and washed with sterile isotonic solution. The cryogel sheets were cut into 1 × 1 cm square pieces, additionally decontaminated with penicillin (5 kU/mL)/streptomycin (5 mg/mL) solution and equilibrated with excess of sterile isotonic solution. The studied materials were aseptically implanted into subcutaneous pockets so that the control (non-doped) and metal-doped cryogels (TM concentration = 0.2 mM) were alternately placed at the right and left sides of an animal. The procedure allowed us to decrease the number of animals per group to *n* = 6. No manifestations of pain, infection or any worsening of animal behavior were observed during the experiment.

### 2.9. Histological Evaluation

On days 5 and 10 post-implantation, the animals were sacrificed using tiletamine-zolazepam-xylazine anesthesia and by applying incremental concentration of CO_2_. The treated skin with implanted material was surgically excised, then subsequently fixed in 4% neutral buffered formalin solution in PBS at room temperature for 48 h, washed with distilled water, dehydrated in a graded series of ethanol solutions (50, 70, 90, 96, 99.8%) and cleared in xylene. The explants were embedded in paraffin blocks and cut on a microtome HM 355S (Thermo Fisher Scientific) into 10–14 μm sections. The tissue sections were stained with Giemsa, hematoxylin and eosin (H&E) or Picrosirius red and analyzed by bright-field and polarized light microscopy on an Axio Observer Z1 microscope (Carl Zeiss). 

For immunohistochemical analysis, glass slide-adhered tissue sections were permeabilized using 0.1% Triton X-100 in PBS for 30 min, thoroughly washed with PBS, and blocked with 1.5% BSA. The sections were subsequently incubated with primary antibodies (diluted 1:300 in 1.5% BSA/PBS) against CD-31 (PECAM-1), HIF-1α, MMP-2 or MMP-3 overnight at 4 °C followed by incubation with proper Alexa Fluor 488-conjugated donkey anti-goat and Alexa Fluor 647-conjugated donkey anti-mouse secondary antibodies (1:350 in 1.5% BSA/PBS), for 45 min at room temperature. DAPI was used to stain cell nuclei. LSCM images were acquired on an LSM 780 microscope.

### 2.10. Statistical Analysis

Data were presented as mean ± SD. Statistical significance was determined by one-way analysis of variance (ANOVA) followed by Tukey’s Multiple Comparison post-test (* *p* < 0.05, ** *p* < 0.01, *** *p* < 0.001).

## 3. Results

### 3.1. Characterization of Cryogels

Cryogels were made by the cryotropic gelation of bovine gelatin cross-linked with glutaraldehyde. Co^2+^, Cu^2+^ and Zn^2+^ (further designated as Co, Cu and Zn) in the form of water-soluble salts were incorporated into the cryogel material during gelation. The addition of pectin to the gelatin cryogel has been used to facilitate the capture of metal ions by introducing additional anionic groups, such as galacturonic acid, into the polymer network. The metal content in the cryogels linearly depended on the concentration of TMs in the gel-forming solution from 0.04 to 5 mM; the TM-doped cryogels were designated by these concentrations. For the upper 1 mM concentration used in this study, the TM content in cryogels was previously shown to amount to ca. 3 × 10^3^ ppm (Zn, Cu) and 1 × 10^3^ ppm (Co) [19].

**Figure 1:**
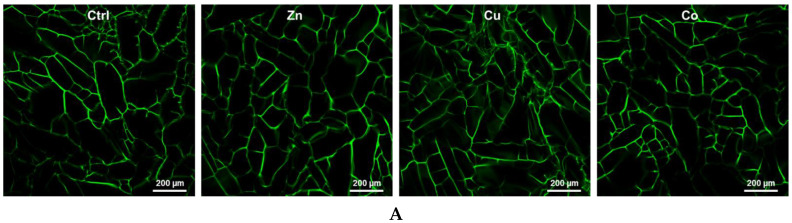

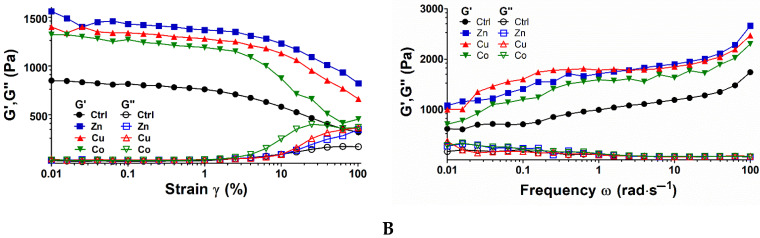
(**A**) LSCM images of non-doped (Ctrl) and TM-doped cryogel sheets (top surface) visualized by autofluorescence upon argon laser excitation (488 nm). (**B**) Strain amplitude sweep test (angular frequency ω = 10 rad s^−1^) and frequency sweep test (strain deformation δ = 1%) data for the cryogels. TM-doped cryogels (1 mM) were analyzed.

According to LSCM analysis, the TM-doped cryogels preserved an interconnected porous structure (Figure 1A), typical of cryogel scaffolds, with somewhat larger pores at the upper surface, commonly used for cell culture [34]. Non-doped cryogels possessed macropores with a calculated mean pore size of 80 ± 13 μm, which moderately decreased by 1.2–1.4 times in the TM-doped materials. According to rheological data, all cryogels displayed a linear viscoelastic region for a shear strain amplitude of γ ≤ 6% (Figure 1B). Both storage (G′) and loss (G″) modulus relatively weakly depended on angular frequency, whereas G′ greatly prevailed over G″ (Figure 1B), indicating a well-structured hydrogel network with dominant elastic behavior. The incorporated TMs increased the G′/G″ ratio by 1.3–1.9-fold, with a relatively lower effect of Co, thus demonstrating that the metal component contributes to the elasticity and mechanical strength of the materials. The modulation of pore size and viscoelastic behavior of cryogels by the introduced TMs is explained by additional cross-linking of macromers (gelatin and pectin molecules) by the metal ions presumably involving not only ionic but also coordination bonding with biopolymer ligand groups.

### 3.2. Behavior of Fibroblasts in Metal-Doped Cryogels

#### 3.2.1. Effect of Cryogel Composition on Cell Proliferation

**Figure 2:**
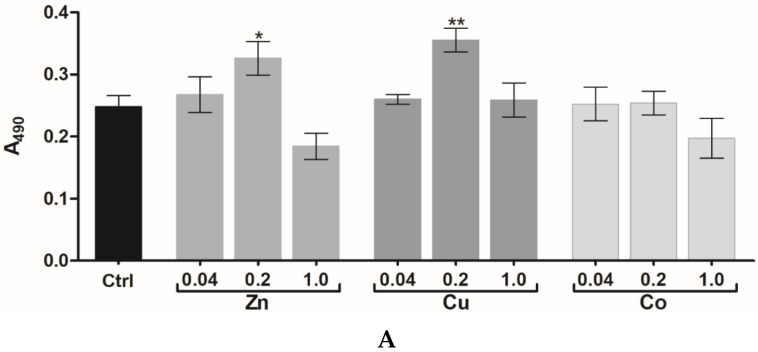

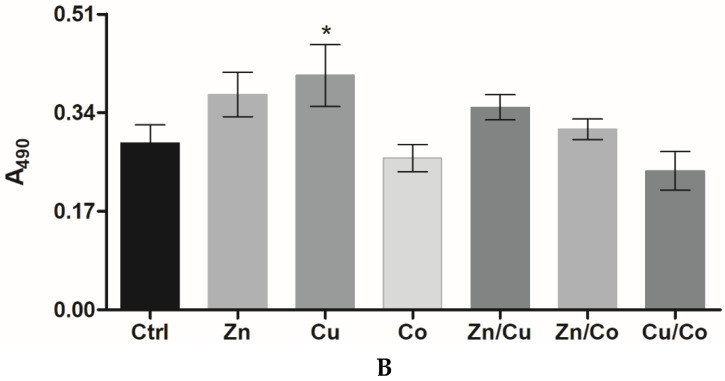
(**A**) Proliferation of 3T3 fibroblasts in TM-doped cryogels at different metal concentrations (MTS assay, 72 h). (**B**) Effect of metal dopants (0.2 mM) on HSF proliferation in the cryogels (MTS assay, 72 h). The data are presented as mean ± SD (*n* = 3, * *p* < 0.05, ** *p* < 0.01).

Mouse embryonic fibroblasts (3T3 cells) were seeded on the top surface of cryogels and allowed to grow for 72 h followed by cell detection using the MTS proliferation assay [34]. At a concentration of 0.04 and 0.2 mM, the introduced TMs did not inhibit cell proliferation, whereas 1 mM Co and Zn, unlike 1 mM Cu, caused a moderate inhibitory effect of up to 24% (Figure 2A). When supplemented into the culture medium, soluble TMs had IC_50_ values of 170 ± 9 µM (Zn), 250 ± 17 µM (Co), and 407 ± 20 µM (Cu) (72 h). Assuming most of the amount of TMs in gelling solution to be attached to the cryogel [19], the above data together suggest that the entrapped metals are not readily released into the medium remaining less available and less cytotoxic toward the cells than dissolved metals. In total, 0.2 mM of Cu and 0.2 mM of Zn were found to noticeably stimulate cell proliferation, respectively, by 30 and 10% (Figure 2A); this intermediate concentration was, therefore, selected for further study and comparison of regenerative effects of the TM-doped cryogels.

**Figure 3:**
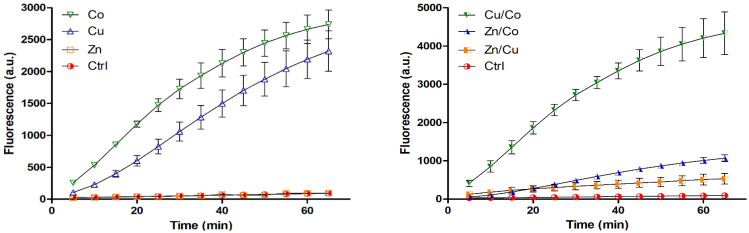
Generation of ROS in reaction of TM-doped cryogels with H_2_O_2_ according to DCFDA fluorescence (λ_ex_ = 490 nm, λ_em_ = 526 nm).

Similar to 3T3 cells, human skin fibroblasts (HSFs) proliferated more rapidly in the cryogels with 0.2 mM Zn (by 29%) or Cu (by 40%) (*p* < 0.05), unlike Co (Figure 2B). Furthermore, binary TM compositions exhibited quite different effects on HSF behavior. In particular, Zn/Cu did provide additive stimulation of cell growth compared to the individual metals, whereas the stimulating effect was partially preserved for Zn/Co and disappeared for Cu/Co system (Figure 2B). Considering that such a mitogenic activity could be associated with Fenton-like reactions of TMs [29], the ability of cryogels to generate ROS in the reaction with hydrogen peroxide (H_2_O_2_) was analyzed with the aid of a DCFDA probe (Figure 3). The results show that the cryogel-formulated metal dopants are capable of reacting with H_2_O_2_, where Co and Cu, unlike Zn, effectively generate ROS (Co > Cu) in agreement with earlier observation for these TMs’ behavior in solution [35]. When introduced together, Co and Cu showed additive prooxidant activity, whereas the individual activity of Co or Cu was profoundly inhibited in the presence of Zn co-dopant, reflecting its antioxidant/anticorrosive effect toward the metals with variable valency [28].

#### 3.2.2. Cytokine and Growth Factor Profile

**Figure 4:**
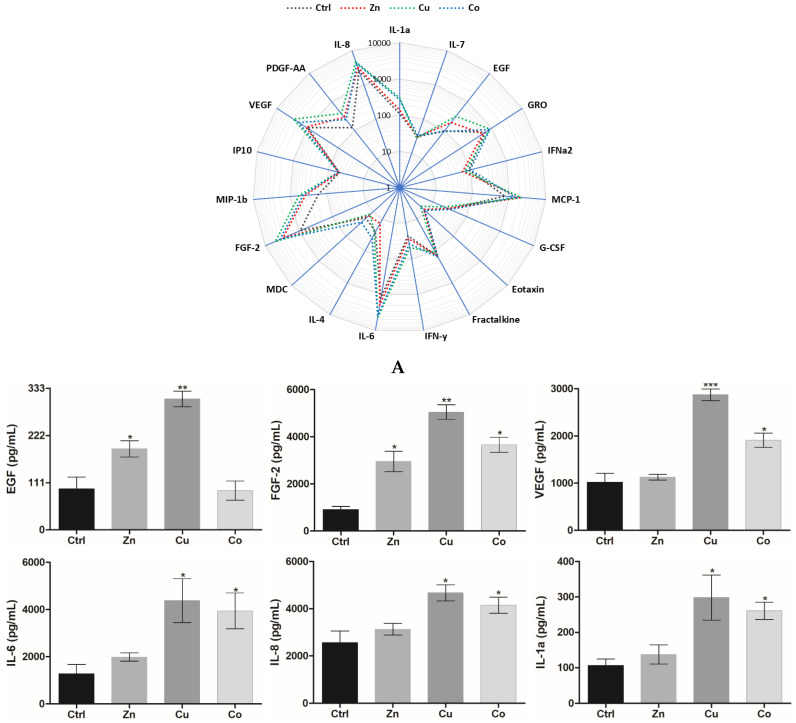

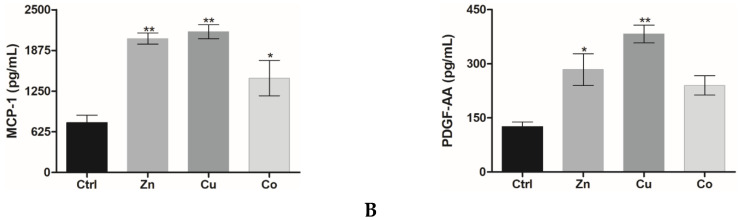
(**A**) Radar plot representation of secreted cytokine/growth factor profile for HSFs cultured in TM-doped cryogels (0.2 mM) for 24 h (pg/mL, log scale). (**B**) The corresponding levels of selected cytokines/growth factors. The data are presented as mean ± SD (*n* = 3, * *p* < 0.05, ** *p* < 0.01, *** *p* < 0.001).

The effect of metal dopants on the production of cytokines and growth factors by HSFs in the cryogels was assessed (Figure 4A). The secretion of FGF-2, VEGF, IL-6, and IL-8 tightly involved in paracrine stimulation of angiogenesis [26] was profoundly stimulated (by 1.2–5.6 times relative to the non-doped cryogel) generally as follows: Zn < Co < Cu (Figure 4B). Monocyte chemoattractant proteins (MCP-1, MIP-1b) were also overproduced in the presence of TMs. In comparison with Cu and Co, Zn weakly affected the level of pro-inflammatory cytokines IL-6/IL-8, whereas it greatly increased the level of MCPs comparably with Cu and Co. The TMs induced the overproduction of EGF and PDGF-AA, similar to other pleiotropic growth factors, namely, FGF-2 and VEGF (by 1.9–3.2 times). Exceptions were that Co and Zn exhibited a lack of effect, respectively, in the case of EGF and VEGF (Figure 4B). The results demonstrate a strong ability of the cryogel-formulated TMs to increase key signaling molecules involved in regeneration-related processes.

### 3.3. Behavior of HUVECs in Metal-Doped Cryogels

#### 3.3.1. Proliferation and Spreading

**Figure 5:**
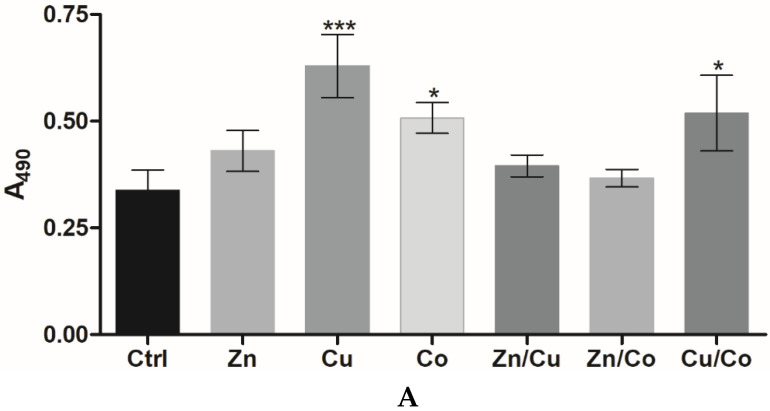

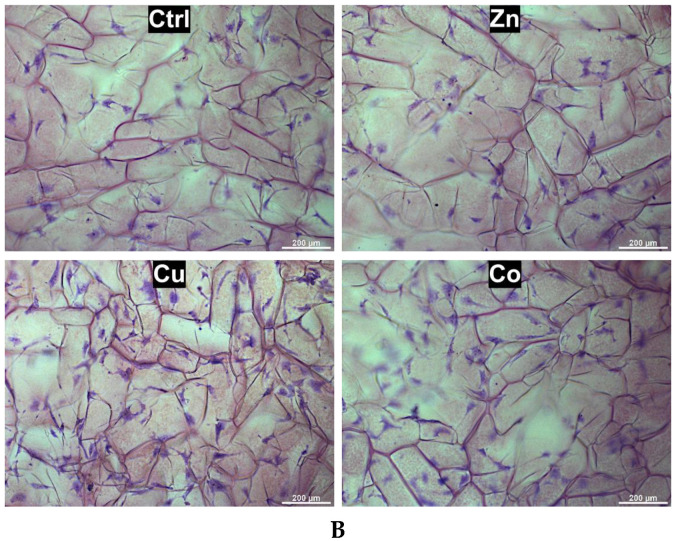
(**A**) Effect of metal dopants (0.2 mM) on proliferation of HUVECs cultured in TM-doped cryogels (MTS assay, 72 h). The data are presented as mean ± SD (*n* = 3, * *p* < 0.05, *** *p* < 0.001). (**B**) Corresponding bright-field microscopy images of HUVECs stained with cresyl violet in metal-free (Ctrl) and TM-doped cryogels.

Human umbilical vein endothelial cells (HUVECs) were used as reference endothelial cells (EC) to compare angiogenic properties of the TM-doped cryogels. The metal dopants (0.2 mM) did not cause any inhibition of HUVEC proliferation, similar to that observed for HSFs, confirming the cytocompatibility of the materials for primary human cells. Furthermore, HUVECs proliferated much faster in the presence of TMs by a factor of ca. 1.3 (Zn), 1.5 (Co) and 1.9 (Cu) compared to the control cryogel (Figure 5A). In comparison with HSFs, HUVECs were characterized by higher sensitivity to Cu and Co, whereas Zn almost abolished the stimulating effect of Cu and Co in binary compositions (Figure 5A), presumably in relation to the ROS-modulating activity of the TMs (Figure 3).

In addition, HUVECs were visualized within the semi-transparent cryogels after cell staining with cresyl violet (Figure 5B). The calculated cell number per 1 mm^2^ of the analyzed surface was as follows: 47 ± 8 (Ctrl), 53 ± 6 (Zn), 102 ± 16 (Cu) and 73 ± 11 (Co), supporting the fact that the corresponding MTS signals (Figure 4A) reflected the cell density in the matrix rather than the change in metabolic activity. Furthermore, HUVECs were well adhered and spread on the surface of all cryogels and their morphology was altered to spindle-shaped cells with developed extensions. Specifically, in the Cu-doped cryogel, up to 41% of the cells adopted more elongated morphology with at least three sprouts; the adjacent cells showed a tendency to migrate and connect to each other, which is attributed to the induction of tubulogenesis.

#### 3.3.2. Angiogenic Differentiation

**Figure 6:**
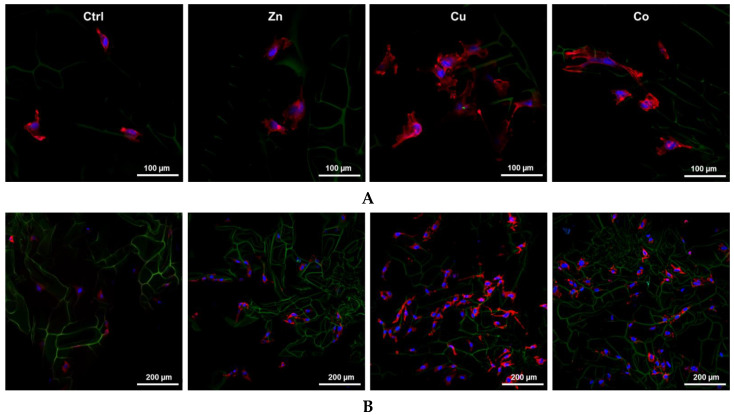

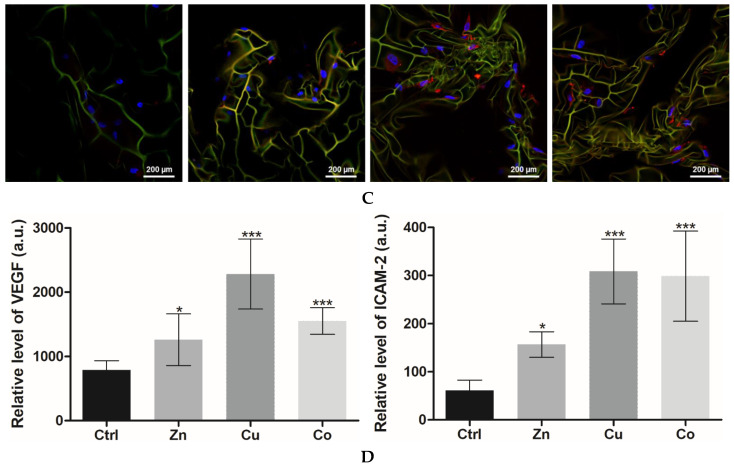
LSCM images of HUVECs grown in TM-doped cryogels (0.2 mM) at day 3 post-seeding. (**A**) Cells stained with phalloidin CruzFluor™ 647 conjugate for F-actin (red). (**B**) Immunofluorescence detection of VEGF. (**C**) Immunofluorescence detection of ICAM-2. Cell nuclei were stained with DAPI (blue). (**D**) Relative density of VEGF and ICAM-2 expression per field of view (mean ± SD, * *p* < 0.05, *** *p* < 0.001).

The markers of HUVEC differentiation in the TM-doped cryogels were detected by LSCM. In the presence of TMs, phalloidin CruzFluor™ 647-stained cells showed profound morphological changes with reorganization of actin cytoskeleton (Figure 6A).

In the Cu-doped cryogel, the cells were characterized by increased spreading with ca. 2.2-fold bigger area than control cells (mean cell area detected was 5807 ± 506 and 2595 ± 468 μm^2^, respectively). According to immunofluorescence analysis, the expression of VEGF and ICAM-2 factors, which regulate EC viability, migration and microvasculature formation [36], was increased by the metal dopants as follows: Zn < Co < Cu, approximately 1.6–2.9-fold for VEGF and 2.6–5-fold for ICAM-2 (Figure 6B,C). This further suggests profound angiogenic activity of the TMs, especially Cu, under experimental conditions.

### 3.4. Effects of Metal-Doped Cryogels upon Subcutaneous Implantation

#### 3.4.1. In Vivo Model Overview

**Figure 7:**
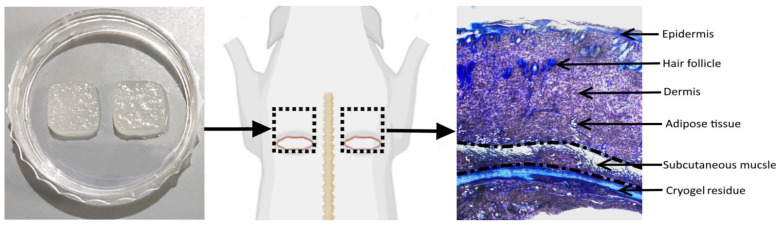
Schematic representation of implantation of cryogel sheets (1 × 1 cm) into subcutaneous pockets (1.5 × 1.5 cm, dotted line) in rats and main skin structures subjected to histological analysis. See Section 2.8.2 for details.

Based on previous surgical procedures [30,31,32], subcutaneous implantation model in Wistar rats was optimized to assess well-defined effects of the cryogel-formulated TMs. Two subcutaneous square pockets (1.5 × 1.5 cm) were formed at the upper dorsal surface by means of incision at the lower side of the outlined square zone followed by the skin detachment from underlying tissues (Figure 7). Square cryogel sheets (1 × 1 cm) were aseptically inserted into the formed pockets followed by skin suturing. In comparison with skin excision, the model allows for informative analysis of localized effects of the materials on intact host tissues upon biodegradation and release of TMs. Furthermore, early-stage host tissue responses to the TM-doped cryogels at days 5 and 10 after implantation were selected to better compare regeneration and inflammation-related processes. The main skin layers and appendages from the subcutaneous muscle to the epidermis were histologically examined (Figure 7). The analysis of non-doped and TM-doped materials in the same animal enabled improved assessment of specific activities of TMs, as immune and regenerative responses in rats are intrinsically variable [37], allowing one to reduce the number of animals in each group.

#### 3.4.2. Biodegradation of Cryogels

**Figure 8:**
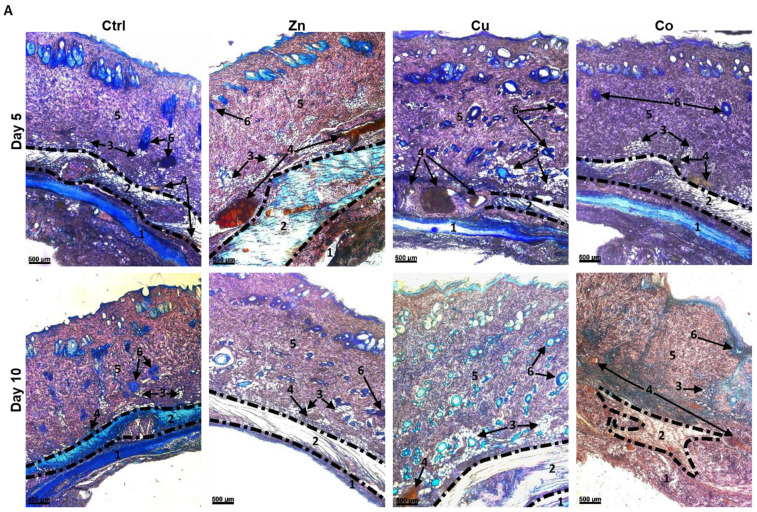

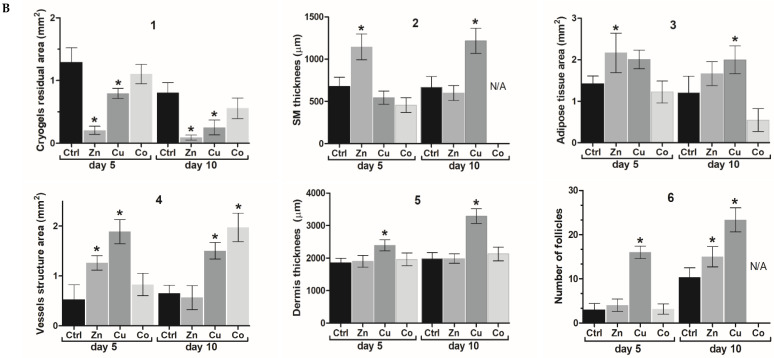
(**A**) Bright-field microscopy images of Giemsa-stained cross-sections of skin explants contacted with subcutaneously implanted TM-doped cryogels. (**B**) Mean morphometric parameters of the treated skin (designated as numbers from **1** to **6**) per cross-section (mean ± SD, * *p* < 0.05).

Lateral sections of the skin in contact with the cryogel were stained with Giemsa or Hematoxylin-eosin; the former stain was selected for histological differentiation between the main skin structures designated as numbers from **1** to **6** (Figure 8). Biodegradation of the implanted cryogels was analyzed by quantifying their residual area (Figure 8, **1**). The detected amount of the non-doped cryogel was reduced by ca. 38% from day 5 to day 10 post-implantation, suggesting initial resorption kinetics of the material. The components Zn and, to a lesser extent, Cu increased the rate of degradation of the cryogel, whereas Co had a relatively weak effect on the process. When the Zn dopant was used, ca. 80% of the cryogel implant was degraded at day 5, whereas comparable degradation was achieved for Cu at day 10 (Figure 8B, **1**). The acceleration of the biodegradation of Zn- and Cu-doped materials could be attributed to the TM-mediated increase in the catalytic activity of matrix metalloproteinases (MMP) [38].

#### 3.4.3. Subcutaneous Muscle and Adipose Tissue

The subcutaneous muscle (SM) and adipose are well-vascularized tissues that host different specialized and multipotent cells and play important roles in cell recruiting, nutrition, biosynthesis of extracellular matrix (ECM) and angiogenesis [39,40]. At day 5, the Zn dopant induced a profound 1.7-fold increase in the thickness of SM (Figure 8, **2**), which is in immediate contact with the cryogel, whereas this parameter was weakly affected by Cu and showed a tendency to decrease in the presence of Co. At day 10, the stimulating effect of Zn on SM decreased, probably in relation to rapid resorption of the Zn-doped cryogel, whereas Cu exhibited comparable stimulation to Zn at day 5. Furthermore, in the presence of Zn and Cu dopants, SM was reorganized so that round rolled muscle structures became unrolled; the latter SM configuration favors cell migration and proliferation upon skin regeneration [40]. Furthermore, SM was greatly disrupted and mostly replaced by an amorphous connective tissue when the Co-doped cryogel was applied (day 10), complicating corresponding analysis (Figure 8B, **2**).

Adipose tissue (Figure 8, **3**) detected as hollow reticular areas (corresponding to washed-out lipid contents of adipocytes) [41] was also enlarged and deeply penetrated into the dermis in the presence of Zn and Cu components, respectively, by ca. 1.5 times (day 5) and over 1.7 times (day 10), compared to the control (non-doped cryogel). This effect of TMs was observed along with morphological change of some adipocytes from round- to ellipsoid-shaped cells. In great contrast to Cu and Zn, Co generally disrupted the adipose structures by day 10 (Figure 8B, **3**).

#### 3.4.4. Vascular System

In comparison with the control material, the Zn- and Cu-doped cryogels did not significantly change the distribution pattern of vascular structures (Figure 8A), which were predominantly localized in adipose and SM tissues, though causing noticeable expansion of these structures (Figure 8B, **4**). At day 5, the skin vascularization assessed by the relative area of the vasculature was increased by the Zn and Cu dopants (Cu > Zn), whereas at day 10 this effect was shown to be dissipated for Zn and maintained for Cu, presumably reflecting resorption properties of the corresponding materials (Zn > Cu). Co, though disintegrating intact SM and adipose layers, at day 10 induced a profound formation of many relatively small vessels in different skin layers, including upper dermis tissues (Figure 8A), which is not typical for intact rat skin. The results suggest all the metal dopants as angiogenic factors as follows: Co ≥ Cu > Zn. This assumes excessive activity of the Co component (also noting decreased degradation of the Co-doped cryogel).

#### 3.4.5. Dermis

The Cu dopant caused a noticeable thickening of the dermal layer (Figure 8B, **5**) by 1.3 and 1.7 times for days 5 and 10, respectively, attributed to extracellular matrix (ECM) overgrowth as a result of Cu-mediated tissue-vascularization. The Zn and Co dopants insignificantly impacted this parameter (Figure 8). Furthermore, the relative area of mature collagen in the dermis was quantified using polarization microscopy with Picrosirius red (Figure S4), which stains mature (type I) and immature (type III) collagens in yellow-red and green colors, respectively [42]. The parameter increased as follows: Co ≤ Ctrl < Zn < Cu, as if the Zn and Cu dopants enhanced collagen maturation by ca. 1.3 and 1.6 times, respectively. These data show the increased growth-promoting effect of Cu over Zn toward the dermis. Co, weakly affecting the thickness and collagenization of the dermis, prompted certain disorganization of its intact fibrillar structure, though to a lesser extent than that observed for SM and adipose tissues (Figure 8A), suggesting a specific distance-dependent disturbing effects of the Co dopant on surrounding tissues.

#### 3.4.6. Hair Follicles and Epidermis

At day 5, the Cu-doped cryogel induced a noticeable increase in the number of hair follicles (**6**) in the dermis, which was 5-fold higher than in the control group, whereas the Zn-doped cryogel approached comparable effect at day 10 (Figure 8A). Most follicles in the Cu and Zn groups contained dermal papillary cells and had defined intensively stained outer covering, which is characteristic of the anagen phase [43,44]. The results show that the Cu and Zn dopants have folliculogenic activity (Cu > Zn), and the effect of Zn seems to be delayed, taking into account the rapid resorption of the corresponding matrix. The Co dopant demonstrated a lack of significant stimulation of folliculogenesis.

The skin treated with both the Cu- and Zn-doped cryogels was characterized by a well-structured epidermis, similar to that of the control group but with a somewhat more developed stratum spinosum layer and increased keratinization (Figure S3). In addition, the Cu dopant noticeably promoted epithelial invagination (Figure S3), which is involved in re-epithelization and folliculogenesis [45]. Under the same conditions, the Co-doped cryogel here and there disturbed the epidermis structure (Figure S3) presumably in relation to the disorganization of underlying skin layers.

#### 3.4.7. Immune Cells Appearance

**Figure 9:**
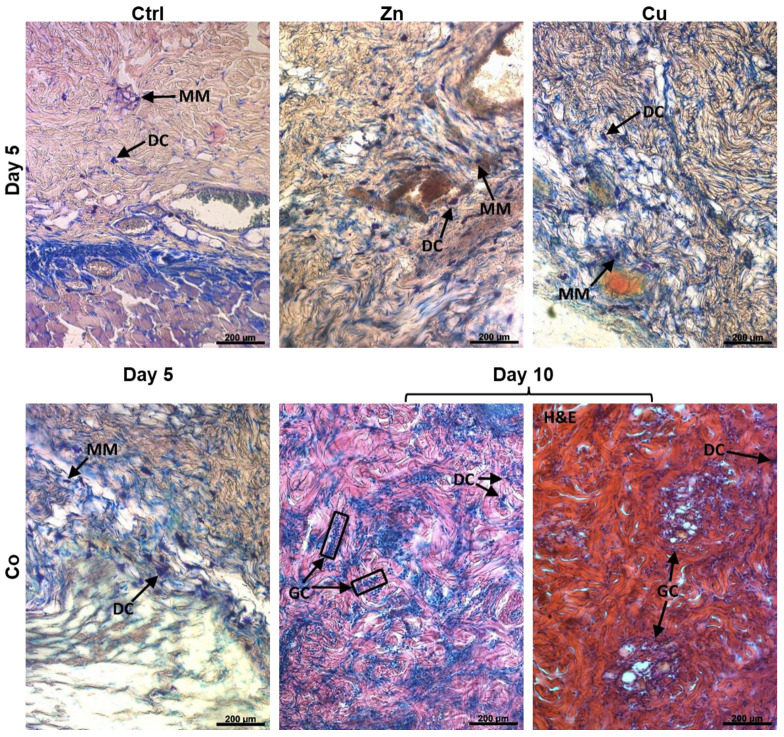
Representative bright-field microscopy images of Giemsa-stained cross-sections of skin explants contacted with subcutaneously implanted TM-doped cryogels. Mononuclear macrophages (**MM**), dendritic cells (**DC**), and multinucleated giant cells (**GC**) were identified. H&E-stained section was additionally shown in right image of lower panel.

The TM-doped cryogels differently affected the appearance of immune cells with distinct morphological features [46] throughout the whole skin. In particular, large intensively stained cells were attributed to mononuclear macrophages having smoothly shaped elongated or roundish morphology with their interior predominantly occupied by a large nucleus (Figure 9, MM) and dendritic cells, which are irregularly shaped due to noticeable cytoplasmic extensions (Figure 9, DC). Furthermore, multinucleated giant cells usually formed as a result of macrophages fusion appeared as huge elongated cells with multiple nuclei [47] (Figure 9, GC).

Upon treatment with the non-doped and Zn-doped cryogels, mononuclear macrophages prevailed over dendritic cells, though the latter material significantly increased overall immune cell number at day 5 (Figure 9). Unlike the above materials, the Cu-doped cryogel (days 5 and 10) and the Co-doped cryogel (day 5) induced a predominant appearance of dendritic cells over macrophages. Furthermore, the Cu group was characterized by a somewhat decreased number of immune cells compared to the Zn and Co groups. The Co dopant in a specific manner resulted in the appearance of numerous giant cells in the dermis at day 10. The provided histological data (Figure 8 and Figure 9) were additionally supported by the corresponding analysis of H&E-stained skin sections (Figure S2). In particular, the latter analysis of the Co group revealed that giant cells are closely located to small capillary structures filled with erythrocytes attributed to newly forming vessels (Figure 9, H&E).

#### 3.4.8. Immunohistochemical Analysis

**Figure 10:**
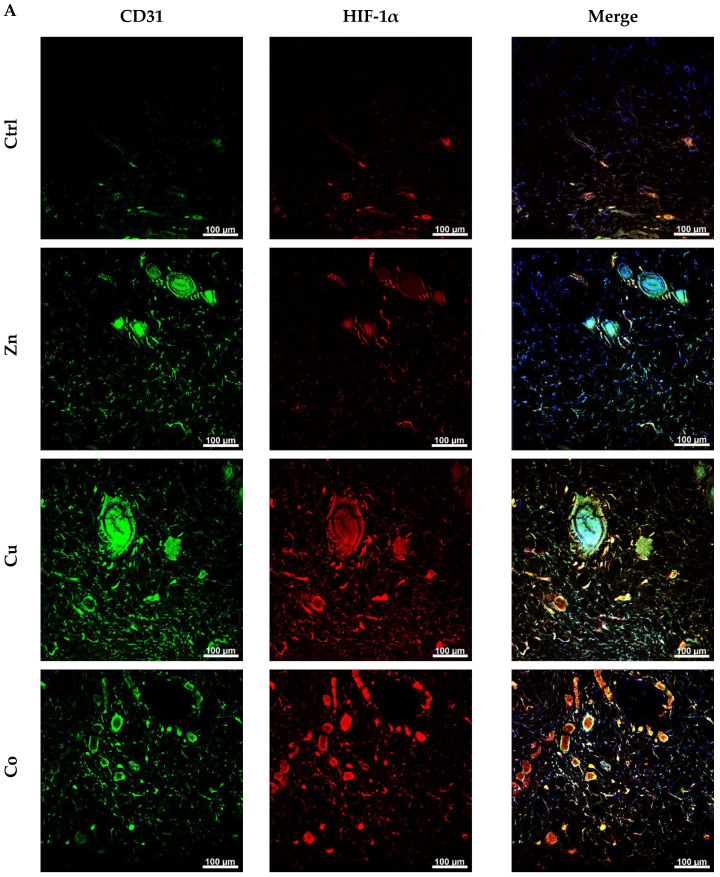

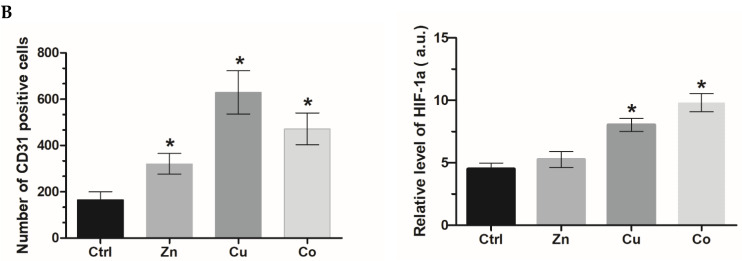
(**A**) Immunofluorescent analysis of cross-sections of skin explants contacted with subcutaneously implanted TM-doped cryogels. Simultaneous CD31 Alexa Fluor 488 (green), HIF-1α Alexa Fluor 647 (red) and DAPI staining was performed. (**B**) Mean number of CD31-positive cells (**left**) and relative density of HIF-1α expression (**right**) per field of view (mean ± SD, * *p* < 0.05).

Additional immunohistochemical analysis of the treated skin (Figure 10 and Figure S5) showed that all TM-doped cryogels increased the number of CD31-positive cells attributed to ECs by a maximal factor of 3.8 (Cu) and 1.9 (Zn) observed at day 5 and 2.9 (Co, day 10) (Figure 10B). These data are consistent with the angiogenic activity of the metal dopants in vivo according to the histological analysis (Figure 8 and Figures S2–S4). Furthermore, the TMs differently affected the production of HIF-1α as follows: Ctrl ≤ Zn < Cu < Co. These data show the ability of prooxidant Co and Cu dopants to induce a hypoxia-like state in the surrounding tissues, which apparently underlies their enhanced angiogenic effects, whereas the Zn component somewhat increased the appearance of ECs and vascular structures without a significant overproduction of HIF-1α.

Furthermore, the Zn- and Cu-doped cryogels were found to significantly increase the dermal level of MMP-2 and MPP-3 involved in the degradation of ECM proteins, the release of ECM-bound growth factors, angiogenesis and tissue remodeling. Similarly, MMP-9 level was elevated in the presence of these materials (data not shown). The effect of metal dopants decreased at day 10 over day 5, as more clearly observed for the rapidly degrading Zn-doped material (Figure S6). The Co-doped cryogel generally did not increase the content of MMPs compared to the control material (Figure S6A,B); however, at day 10, there were distinct structures with highly expressed MMP-2 in the Co group (Figure S6C) attributed to the areas containing giant cells and newly formed capillaries (Figure 9).

## 4. Discussion

The cross-linked gelatin cryogel was used as a biodegradable hydrogel scaffold both to examine regenerative activities of incorporated TMs and to develop improved bioinductive materials. As previously shown, different TMs can be stably incorporated into the cryogel [19], presumably involving complex formation with collagen polypeptide groups in accordance with the earlier observation [48]. The advanced macroporous structure of the cryogels favors their bulk interactions with gases, nutrients and living cells, allowing one to assess regenerative factors in three-dimensional tissue-mimicking conditions [2,34].

Cu, Co, and Zn were studied here as recognized components of solid osteogenic materials [11,12,13,49]; however, therapeutic effects of these TMs in cryogel scaffolds toward soft tissues have not been compared to date. The composition of TM-containing cryogels was optimized so that the metal dopants did not show a cytotoxic effect, while they were able to stimulate the proliferative and functional activity of mammalian cells (Figure 2). The corresponding effective concentrations of the incorporated TMs lay within the range of up to 1 mM, which moderately affected the structure of cryogels (Figure 1). In particular, the metal dopants comparably increased the elastic over viscous behavior of the cryogels up to ca. 2 times (Figure 1B), attributed to additional TM-mediated cross-linking. These data suggest that the incorporated TMs participate in forming a polymer network of the materials and that the stiffness of the TM-doped cryogels should fit with different soft tissues and support cell–matrix interactions [50].

Earlier, the macroporous structure of cryogels allowed us to monitor bulk affinity interactions of the materials with fluorescently labeled peptide ligands [34]. Similarly, the interaction of H_2_O_2_ with TM-doped cryogels was assessed using an ROS-sensitive DCFDA probe, considering H_2_O_2_ as both an extracellular and intracellular precursor of ROS generated in Fenton-like reactions [29,51]. The hydroxyl radical is particularly recognized as a secondary messenger involved in cell redox regulation via oxidizing thiols and activating transcriptional factors such as HIF-1α [52]. The ROS-generating ability of the Co and Cu dopants revealed in the presence of H_2_O_2_ (Figure 3) should reflect hypoxia-mimicking and concomitant angiogenic properties of these TMs [22,24,29]. H_2_O_2_ is known to be released to the site of tissue injury (for example, in association with NADPH oxidase or dual oxidase activities), playing an important regulatory role in the healing process as well as a potential pathological role [53,54]. Therefore, the Co- and Cu-doped cryogels upon tissue implantation are expected to promote the extracellular activity of H_2_O_2_ in contrast to the Zn-doped cryogel.

In comparison with the above reactions (Figure 3), the soluble TMs showed a different ROS-generating profile in 3T3 fibroblasts exposed to 1 or 10 μM compounds in antioxidant-free HBSS (Figure S7). Soluble Cu and, to a lesser extent, Zn, in contrast to Co, were found to induce some ROS overproduction accompanied by a weak decrease in glutathione level in cells. Together, these data support variable condition-dependent ROS-modulating activities of the TMs. The results, in particular, suggest that soluble Zn is also able to increase ROS formation in the fibroblasts, though weaker than soluble Cu (Figure S7), regardless of the ability of Zn co-dopant to inhibit the Fenton-like activity of the Co- and Cu-doped cryogels (Figure 3). The intracellular redox effect of Zn ions can be associated with their interaction with multiple thiol groups of Zn-binding cysteine-rich proteins, particularly metallothionein [55], activation of ROS-producing mitochondrial lipoamide dehydrogenase (LADH) as well as the inhibition of LADH thiol oxidoreductase [56]. Such prooxidant activity is controlled by MTF-1 transcriptional factor activated by increased Zn concentrations, leading to the overexpression of metallothionein and Zn efflux transporters [55,57,58].

The lack of the ROS-generating effect of soluble Co on 3T3 cells (Figure S7) could be explained by the decreased physiological requirement and cellular uptake of this TM in fibroblasts, noting that in cancerous cells the TM readily caused oxidative stress under similar conditions [59]. Unlike soluble Co, nano- and microparticles of this TM were shown to induce oxidative stress in 3T3 cells probably due to the internalization of the particulate formulations by endocytosis and phagocytosis [60]. Interestingly, the proliferation-stimulating effect of the TM-doped cryogels on the fibroblast cells (Figure 2) is in accordance with prooxidant cellular effects of the soluble TMs (Figure S7), suggesting that the former effect involves intracellular uptake of the metal ions released from the materials. Together, our results demonstrate that the studied TMs possess multifaceted ROS-modulating activities depending on the conditions. Variable prooxidant effects of Cu and Co, both extracellular and intracellular, could be particularly expected, whereas Zn is expected to have dual prooxidant/antioxidant effects.

Among the metal dopants, Cu exhibited the highest stimulation of proliferation of all studied cells (3T3 fibroblasts, HSFs, HUVECs) with as high as 2-fold stimulation for HUVECs (Figure 2 and Figure 5). The Co dopant was almost inactive toward 3T3 cells and HSFs, whereas it significantly increased the proliferation of HUVECs, suggesting increased sensitivity of these ECs to the TMs compared to other types of cells in accordance with reported effects of the GHK-Cu complex [20] and metal chelators [61].

Based on the above results, the mitogenic activity of the TM-doped cryogels could be explained by the activation of ROS-mediated signaling pathways in the cells [52]. Such an effect seems to require an appropriate cell-specific level of overproduced ROS, presumably achieved in both fibroblasts (Figure 2) and HUVECs (Figure 5) grown in the Cu- and Zn-doped cryogels. This level should be essential for the stimulation of regenerative processes, particularly angiogenesis [62]. Excessive ROS levels can decrease cell viability and lead to sustained secretion of pro-inflammatory cytokines and endothelial permeability [63]. Therefore, double composition of prooxidant TMs, namely, Cu and Co, demonstrated lower or lack of stimulation toward HUVECs/HSFs compared to Cu alone (Figure 2B and Figure 5A) attributed to excessive generation of cellular ROS. Moreover, the Zn co-dopant abolished stimulation of HUVECs in the Cu- and Co-doped cryogels (Figure 5A) and decreased it for HSFs in the Cu-doped cryogel (Figure 2B). This is attributed to the prevention of ROS production by the Zn co-dopant presumably via the inhibition of the Fenton-like reaction catalyzed by Cu and Co [52] and/or the possible reversal of HIF1-α upregulation induced by the latter TMs [64].

The mitogenic activity of the metal dopants was accompanied by the angiogenic differentiation of HUVECs in the presence of Cu and Co viewed through the morphological rearrangement and overexpression of VEGF and ICAM-2 (Figure 5B and Figure 6). Furthermore, all the TM dopants induced the overexpression of a series of cytokines and growth factors by HSFs (Figure 4), including VEGF, FGF-2 and PDGF, the potent regulators of functional activity of ECs and blood vessels [65]. Moreover, the enhanced release of MCP-1 by the Zn and Cu dopant (Figure 4) may be essential for the recruitment of monocytes/macrophages involved in tissue repair and angiogenesis [66,67]. Of note, the aforementioned cytokines and growth factors can be produced and secreted by ECs, playing important roles in the autocrine regulation and recruitment of other cells to support neovascularization [20]. Thus, our results show that the incorporated TMs allow for the effective induction of pleiotropic growth factors and cytokines in the cryogel-grown cells. Such an activation of cryogels with TMs provides a promising alternative to the immobilization of low-stable recombinant polypeptides such as VEGF [68] and FGF-2 [69], solely or in combination [70], in order to improve regenerative and angiogenic properties of polymeric scaffolds.

The subcutaneous evaluation allows one to understand regenerative or toxicological mechanisms of (bio)materials at molecular, cellular and tissue levels [30,31,32,71,72], and these are relevant not only for skin but for other organs and tissues. The potential of subcutaneous implantation for the analysis of TM-containing bioactive hydrogels is poorly investigated, apart from a few reports [73,74] not dealing with TM comparison. Such a model was optimized here to establish specific localized effects of macroporous cryogel-formulated TMs on various intact skin tissues and appendages (Figure 7).

The degradation rate of implanted TM-doped cryogels was in the order: Zn > Cu > Co ≥ Ctrl (Figure 8, **1**). The profound promoting effect of the Zn dopant is apparently associated with this TM activity as a key cofactor of different MMPs, including MMP-1, MMP-3, MMP-8, MMP-13, MMP-2, and MMP-9 [75]. The peptidase activity of MMP-1 and MMP-9 can be also promoted by Cu [38] in accordance with some lesser effect of the latter dopant. Some delayed effect of the Co dopant at day 10 (Figure 8, **1**) may be attributed to the induction of MMP-1 and MMP-2 expression under oxidative stress conditions [76,77]. Immunofluorescence analysis confirmed a significant increase in the expression of MMP-2 and MMP-3 (as well as MMP-9) in the dermis in the presence of Zn- and Cu-doped cryogels, which, however, had a comparable effect with each other (Figure S6). Therefore, the increased resorption of the former material should be due to the overexpression of MMPs and/or a higher contribution to specific peptidase activity by Zn ions as compared to Cu ions.

The Cu dopant induced a significant thickening of the dermal layer along with an increase in the number of hair follicles recognized as an important source of stem and progenitor cells for skin repair (Figure 8). These data confirm the positive role of this metal in tissue renewal and remodeling in concordance with the reported ability of peptide-complexed Cu (AHK-Cu) [78] and Cu-doped bioactive glass [79] to promote the formation and growth of hair follicles. Such an effect could be mediated by the stimulation of angiogenesis facilitating the migration of follicle progenitor cells to the dermis [79]. A folliculogenic activity of the Zn-doped cryogel detected at day 10 is in agreement with the recently reported effect of a Zn-containing chitosan-PEG hydrogel in a full-thickness skin defect model in mice [80].

Stimulation of collagen deposition by the Cu and Zn dopants may be associated with effective vascularization or increased collagen stabilization by lysyl oxidase in the presence of Cu [81] as well as with early Zn-mediated attraction of fibroblasts that produce collagen, among other ECM components [80]. Considering some disorganization of skin layers by the Co dopant (Figure 8), its effect on skin functioning is assumed to be negative, presumably due to excessive oxidative stress. It has been previously shown that the use of the Co component at an increased concentration in hydrogels reduces cytocompatibility, collagen deposition and slows down wound closure and re-epithelization [18].

The established ability of metal dopants to increase tissue vascularization (Figure 8 and Figure 10B) is well explained by the generation of ROS (Figure 3 and Figure S7) in combination with the increased tissue level of ROS-sensitive HIF-1α (Figure 10B). It was reported that the modification of mesoporous bioactive glasses by Co [22] and Cu [24] promoted neovascularization by inducing hypoxic cascade and the expression of HIF-1α in human bone marrow stromal cells, yet the induction of HIF-1α was not investigated in vivo. Moreover, the incorporation of Co in a gauze calcium alginate hydrogel was shown to stabilize HIF-1α expression, stimulate angiogenesis and accelerate full-thickness skin wound healing in mice [18]. HIF-1α is a major transcription regulator of VEGF [18,82], which is consistent with a considerable boost in VEGF secretion by fibroblasts (Figure 4) and expression by HUVECs (Figure 6) cultured in Cu- and Co-doped cryogels. In addition, metal-induced VEGF and FGF-2 (Figure 4) are known to stimulate the production of MMPs that degrade basement membrane and ECM, allowing ECs to migrate and form sprouts [70].

The results suggest that the prooxidant activity of the TMs may occur via both extracellular and intracellular reactions. The antioxidant effect of the Zn co-dopant upon the induced ROS production (Figure 3) presumably was not manifested under experimental conditions in vivo, since the TM was found to cause a weak increase in HIF-1α level and some angiogenic response, which were inferior to the effect of Cu and Co. Other Zn formulations, namely, zinc oxide (ZnO) and zinc peroxide (ZnO_2_) nanoparticles, embedded into a cross-linked polymeric hydrogel promoted angiogenesis via the generation of ROS, in particular, the onsite production of H_2_O_2_ [83]. The Cu-doped cryogel provided a more physiological vascularization with a predominant hypodermic localization, whereas the Co-doped material, disturbing hypodermic tissues, caused the redistribution of newly formed vessel structures into the dermis. Such a distinct vascularization pattern, however, may result from the degree of prooxidant activity of the TMs (Co > Cu).

Consistent with these results, microvessel growth in the skin was earlier stimulated by Cu-containing hyaluronic acid hydrogel [73] and borate glass microfibers [84]. However, no mechanisms of Cu-mediated angiogenic activity and its comparison with other TMs were provided in these studies. According to the density of CD31-positive cells (Figure 10B), the Cu-doped cryogel should have better angiogenic potential than other proposed Cu-containing materials [73,79,84]. Furthermore, Co-containing collagen/alginate-based hydrogel was earlier demonstrated to exhibit an angiogenic effect on rat mesenchymal stem cells accompanied by the expression of CD31 and VEGF. However, this activity was not significantly affected by the Co component in vivo [85]. Likewise, implanted Co-doped sol-gel bioactive glasses promoted the appearance of blood vessel structures; however, angiogenic effects were not assessed quantitatively [86].

The angiogenic effect of the Zn-doped cryogel without a significant induction of HIF-1α (Figure 8 and Figure 10B) could result from the activation of other pathway(s) implicating increased FGF-2 production (Figure 4), faster material degradation by MMPs promoting cell infiltration, and from the attraction of mononuclear cells (Figure 9). It is noteworthy that the in vivo effects of the Zn-doped cryogel generally decreased on day 10 compared to day 5. This suggests a transient activity of TM-doped cryogels presumably associated with the release of metal ions during degradation so that it disappears after resorption of the material. This also confirms the importance of assessing the effects of TM-doped cryogels in the early stages post-implantation.

In the presence of implanted Zn-doped cryogel, recruitment of mononuclear cells was observed (Figure 9). This process is known to be mediated by MCP-1 chemokine [87], which was stimulated by the material in vitro (Figure 4). It was reported the role of Zn in stimulating the infiltration of monocytes into the damaged tissues, where they can differentiate into macrophages [87], and regulating the transition of macrophages from pro-inflammatory to immune-regulatory tissue repair phenotypes [88]. Recruited macrophages can contribute to the angiogenic process via the degradation of ECM leading to EC migration, the release of angiogenic cytokines, and vessel wall formation by differentiating into ECs [66,67]. The incorporation of Zn into the hydroxyapatite-collagen scaffold promoted osteogenesis and angiogenesis by activating the p38 MAPK signaling pathway in the monocytes, further contributing to the release of TGF-β, VEGF, and PDGF, which stimulate the recruitment of BMSCs and ECs to the injury site [89]. The regulatory roles of Zn on immune system homeostasis have been reviewed [90]. These particularly involve a balance between normal immune response and potential tissue damage in relation with Zn distribution in the extracellular and intracellular compartments.

The detected overall number of immune cells in the case of Cu-doped cryogels was lower compared to the other implanted gels (Figure 9), which indicates that the host immune response to this material occurred smoothly. On day 10 post-implantation, the Co dopant caused the formation of multinucleated giant cells surrounding vascularization zones in dermis (Figure 9), in concordance with the reported enhanced angiogenesis by these VEGF-expressing cells within the implantation bed [47]. Subcutaneously implanted Co-containing bioactive glasses presented a significantly increased number of cell nuclei, morphologically resembling the detected giant cells [86]. This was attributed to the HIF-1α-mediated recruitment of proinflammatory cells and coordination of regeneration processes by the material [86]. Given that lymphocytes are essential for giant cell formation [91], it could be assumed that Co may trigger an adaptive immune response, the mechanism of which should be studied elsewhere.

## 5. Conclusions

This study proves biodegradable cryogels as advanced hydrogel scaffolds both to clarify the regeneration-related effects of incorporated TMs in vitro and in vivo and to develop improved biomaterials activated with Cu, Co, Zn or their compositions. Since these effects are assumed to be associated with ROS generation, the ROS-modulating activity of the TMs was characterized. The results suggest Cu and Co as well as their binary compositions as probable extracellular Fenton-like prooxidants, which are antagonized by Zn. All the TMs are capable of intracellular ROS generation to different extents and depending on cellular uptake. The ROS-modulating activities of the TMs and compositions were consistent with the proliferation rate of mammalian cells, the production of regeneration-related cytokines and growth factors by HSFs and the angiogenic differentiation of HUVECs in the cryogels in vitro. The subcutaneous implantation model was optimized to elucidate and compare host tissue effects of TMs containing cryogels. Biodegradation of the materials was promoted mainly by the Zn and Cu dopants, which also induced the overexpression of matrix metalloproteinases, the stimulation of collagen deposition and hair follicle growth. All the TMs, especially Cu and Co, increased the level of ROS-sensitive markers and vascularization, where more physiological vasculature pattern was observed in the case of Cu compared to Co. The latter dopant, unlike Cu and Zn, caused an obvious disturbance in the organization of skin layers along with the appearance of multinucleated giant cells attributed to the excessive prooxidant effect. The results contribute to the understanding of regenerative and adverse activities of hydrogel-formulated TMs and provide the basis for tissue engineering and regeneration applications of TM-containing cryogels.

## Data Availability

The data presented in this study are contained within the article.

## Supplementary Materials

The following supporting information can be downloaded at: https://www.mdpi.com/article/10.3390/gels8020118/s1, Figure S1: (**A**) LSCM images of dual TM-doped cryogel sheets (top surface) visualized by autofluorescence upon argon laser excitation (488 nm). (**B**) Frequency sweep test (strain deformation δ = 1%) data for the cryogels. Figure S2: Representative bright-field microscopy images of H&E-stained cross-sections of skin explants contacted with subcutaneously implanted TM-doped cryogels. Figure S3: Visualization of epidermis of treated skin according to H&E staining. (**A**) Representative bright-field microscopy images for non-doped and TM-doped cryogels. (**B**) Localized area with dermatitis manifestations caused by Co-doped cryogel. Figure S4: (**A**) Representative polarization microscopy images of Picrosirius red-stained cross-sections of skin explants (dermal area) contacted with subcutaneously implanted TM-doped cryogels. (**B**) Relative area of mature collagen per field of view. Figure S5: Immunofluorescent analysis of cross-sections of skin explants contacted with subcutaneously implanted TM-doped cryogels at day 5 (Zn and Cu) and day 10 (Ctrl and Co). Simultaneous CD31 CruzFluor™ 488 (green), HIF-1α Alexa Fluor 647 (red) and DAPI staining was performed. Figure S6: (**A**) Immunofluorescent analysis of cross-sections of skin explants contacted with subcutaneously implanted TM-doped cryogels (MMP-2 Alexa Fluor 488 (green) and MMP-3 Alexa Fluor 647 (red)). (**B**,**C**) Relative MMP levels in the dermis per field of view. (**D**) The area with localized giant cells (MMP-2, Co-doped cryogel). Figure S7: Effect of dissolved metals on relative levels of (**A**) ROS and (**B**) glutathione in 3T3 fibroblasts according to DCFDA (λ_ex_/λ_em_ = 490/526) and monochlorobimane (λ_ex_/λ_em_ = 380/480) fluorescence.
