# Supplementary material for: Regenerative Activities of ROS-Modulating Trace Metals in Subcutaneously Implanted Biodegradable Cryogel
Source: Gels. 2022 Feb 14;8(2):118. doi: 10.3390/gels8020118 (PMC8872170; doi:10.3390/gels8020118)
Supplement: Supplementary file 1 [file gels-08-00118-s001.zip › gels-1578551-supplementary.pdf]

gels

Supplementary Materials

Regenerative Activities of ROS-Modulating Trace Metals in Subcutaneously Implanted Biodegradable Cryogel

Abdulla A. Yergeshov [1†], Mohamed Zoughaib [1†], Rezeda A. Ishkaeva [1], Irina N. Savina [2] and Timur I. Abdullin [1,*]

[1] Institute of Fundamental Medicine and Biology, Kazan (Volga Region) Federal University, 18 Kremlyovskaya St., 420008 Kazan, Russia; abdulla.ergeshov@mail.ru (A.A.Y.) ZMokhamed@kpfu.ru (M.Z.); RezAAhmadishina@kpfu.ru (R.A.I.);

[2] School of Applied Sciences, University of Brighton, Huxley Building, Lewes Road, Brighton BN2 4GJ, UK; I.N.Savina@brighton.ac.uk (I.N.S.)

* Correspondance: tabdulli@gmail.com, timur.abdullin@kpfu.ru (T.I.A.)

† Authors with equal contribution.

A

B

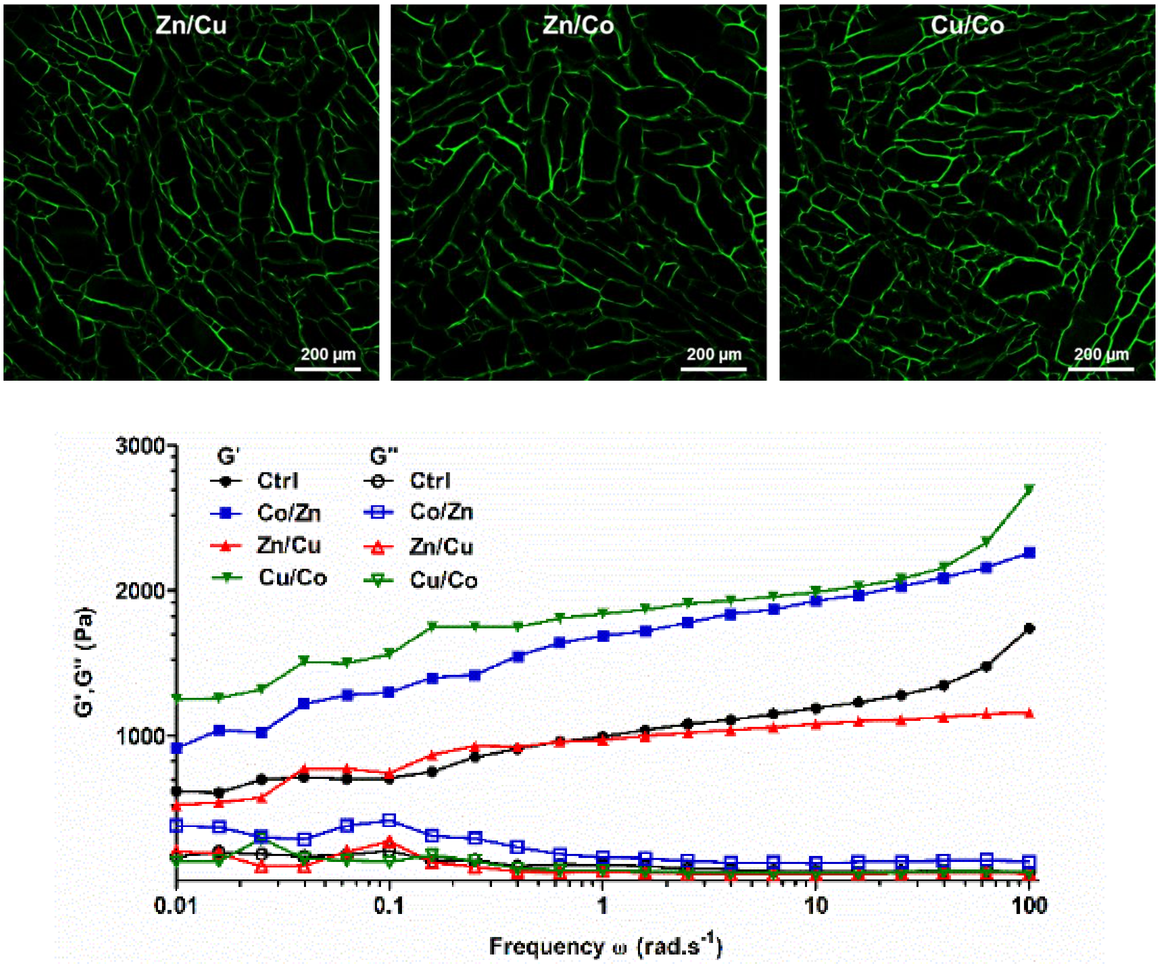

Figure S1. A. LSCM images of dual TM-doped cryogel sheets (top surface) visualized by autofluorescence upon argon laser excitation (488 nm). B. Frequency sweep test (strain deformation δ = 1%) data for the cryogels. TM-doped cryogels (1 mM for each metal) were analyzed.

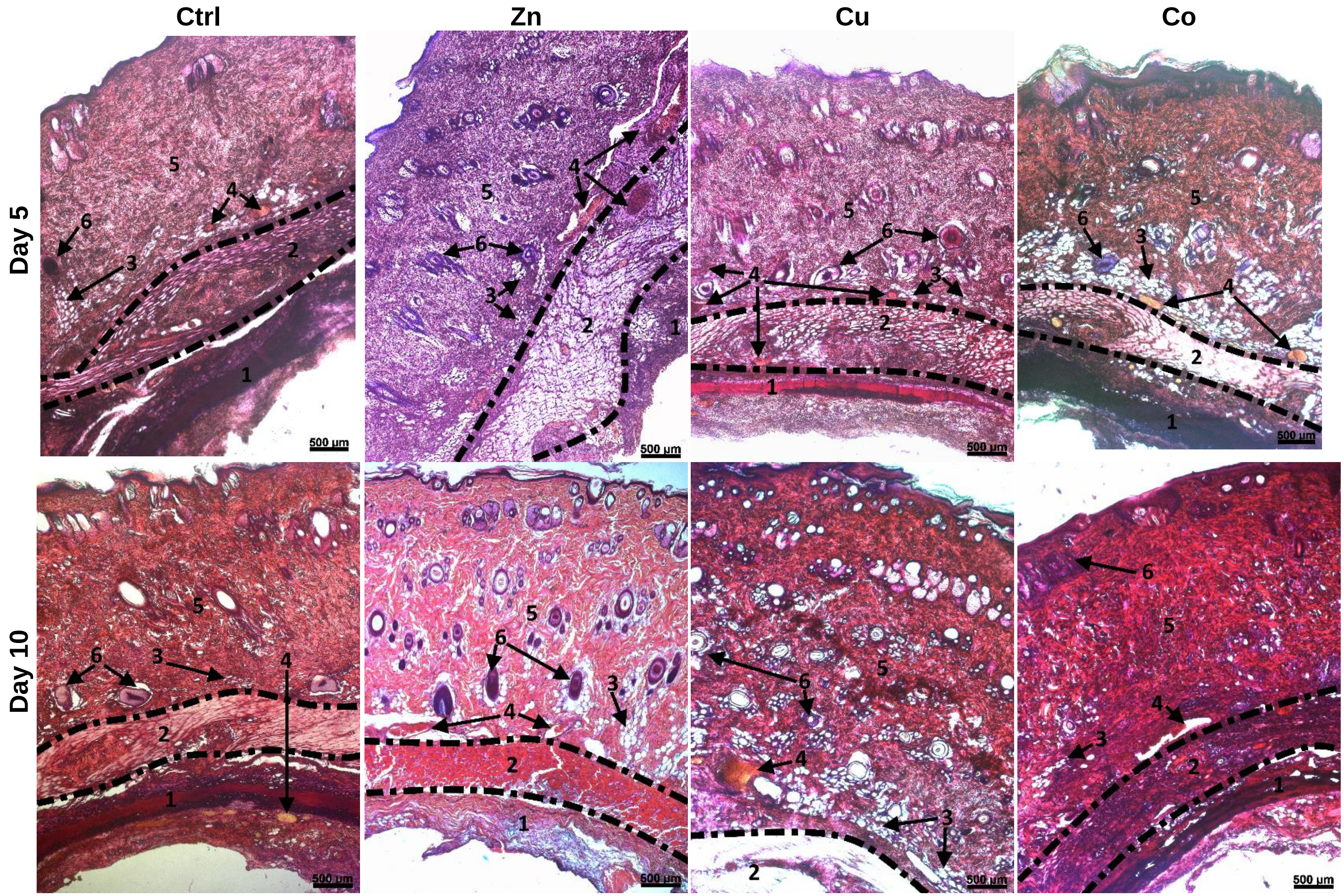

Figure S2. Representative bright-field microscopy images of H&E-stained cross-sections of skin explants contacted with subcutaneously implanted TM-doped cryogels.

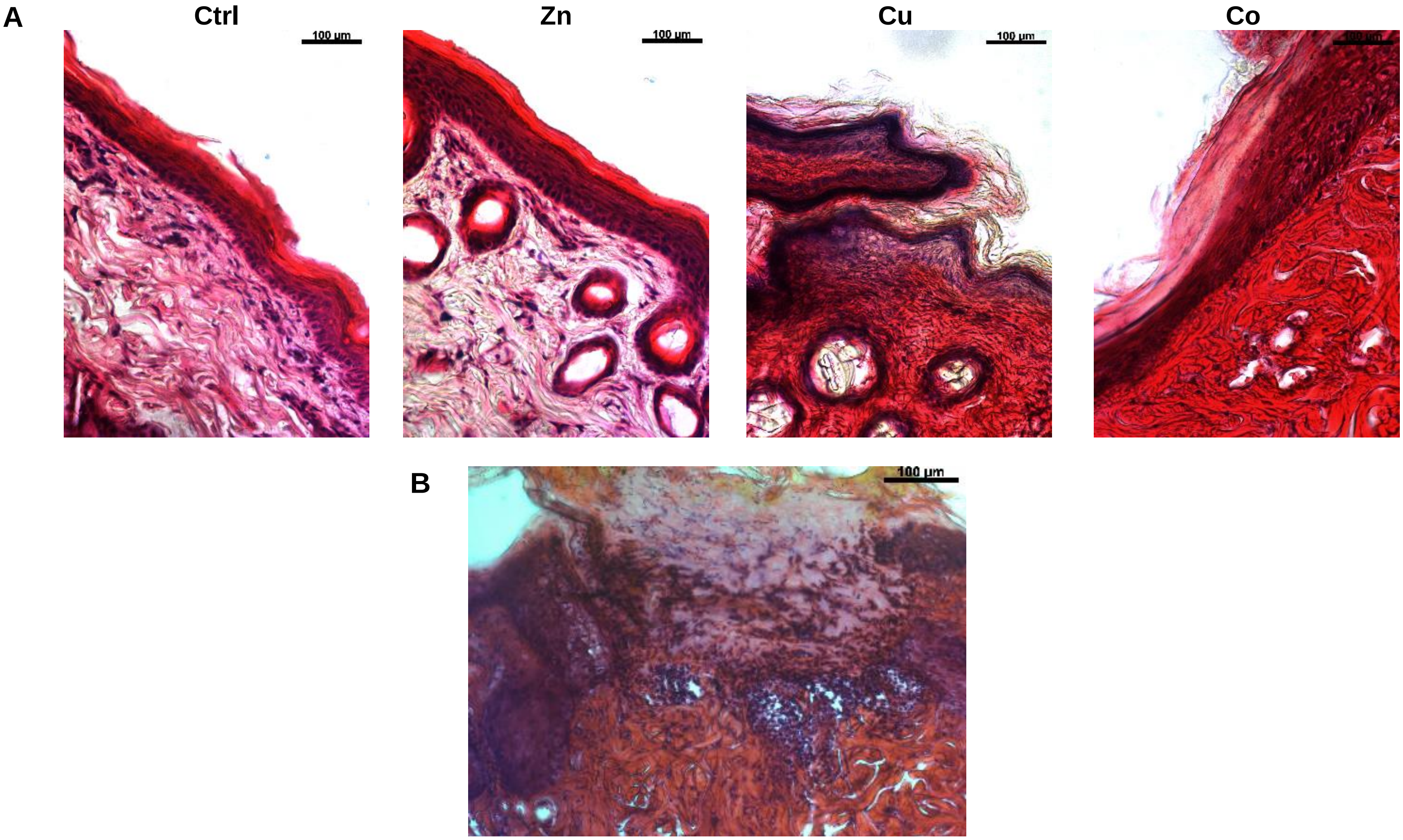

Figure S3. Visualization of epidermal layer of treated skin according to H&E staining. **A**. Representative bright-field microscopy images for non-doped and TM-doped cryogels. **B**. Localized area with dermatitis manifestations caused by Co-doped cryogel.

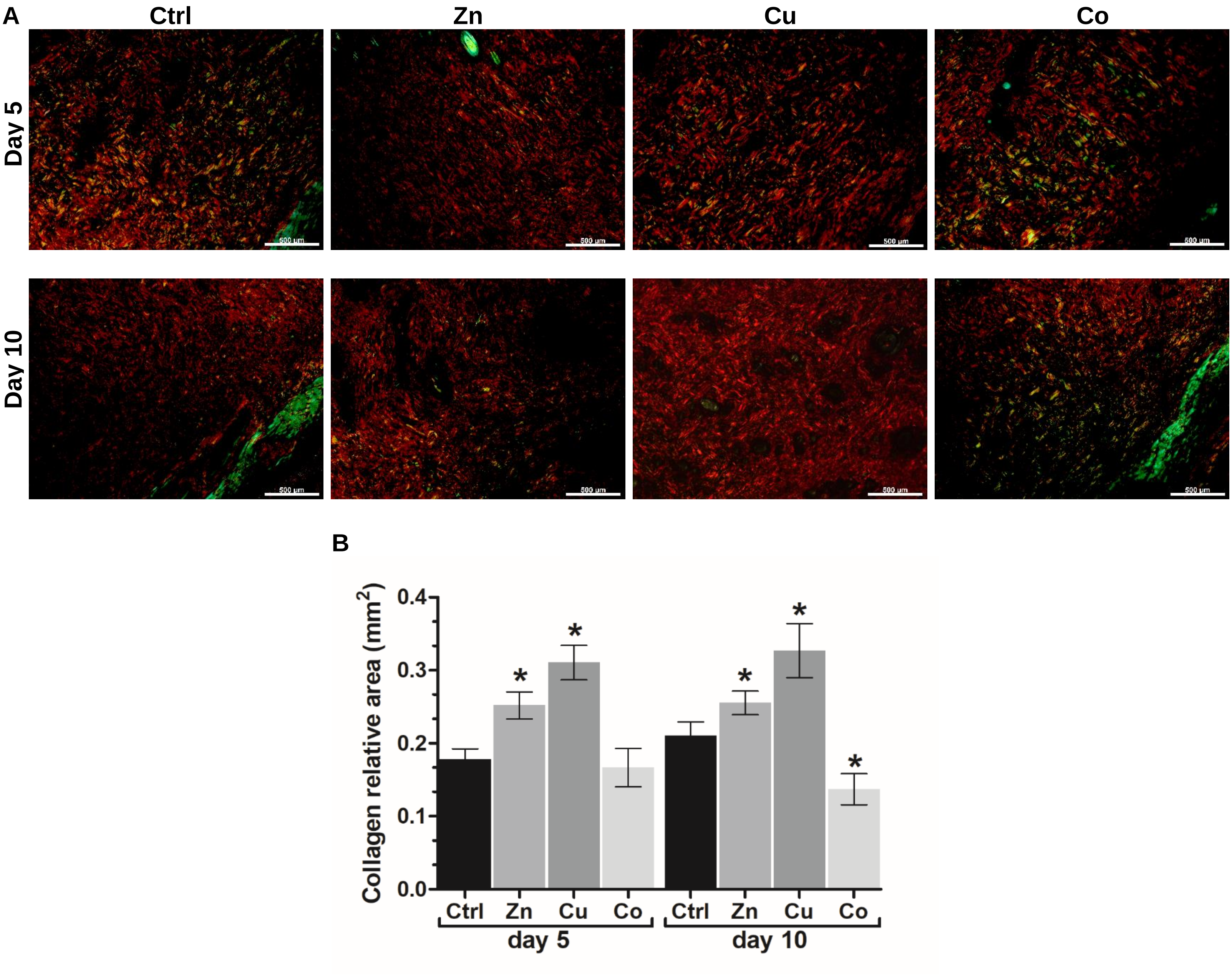

Figure S4. A. Representative polarization microscopy images of Picrosirius red-stained cross-sections of skin explants (dermal area) contacted with subcutaneously implanted TM-doped cryogels. B. Relative area of mature collagen per field of view (mean ± SD, *$p < 0.05$). Red-yellow and green structures, respectively, correspond to mature and immature collagens [41].

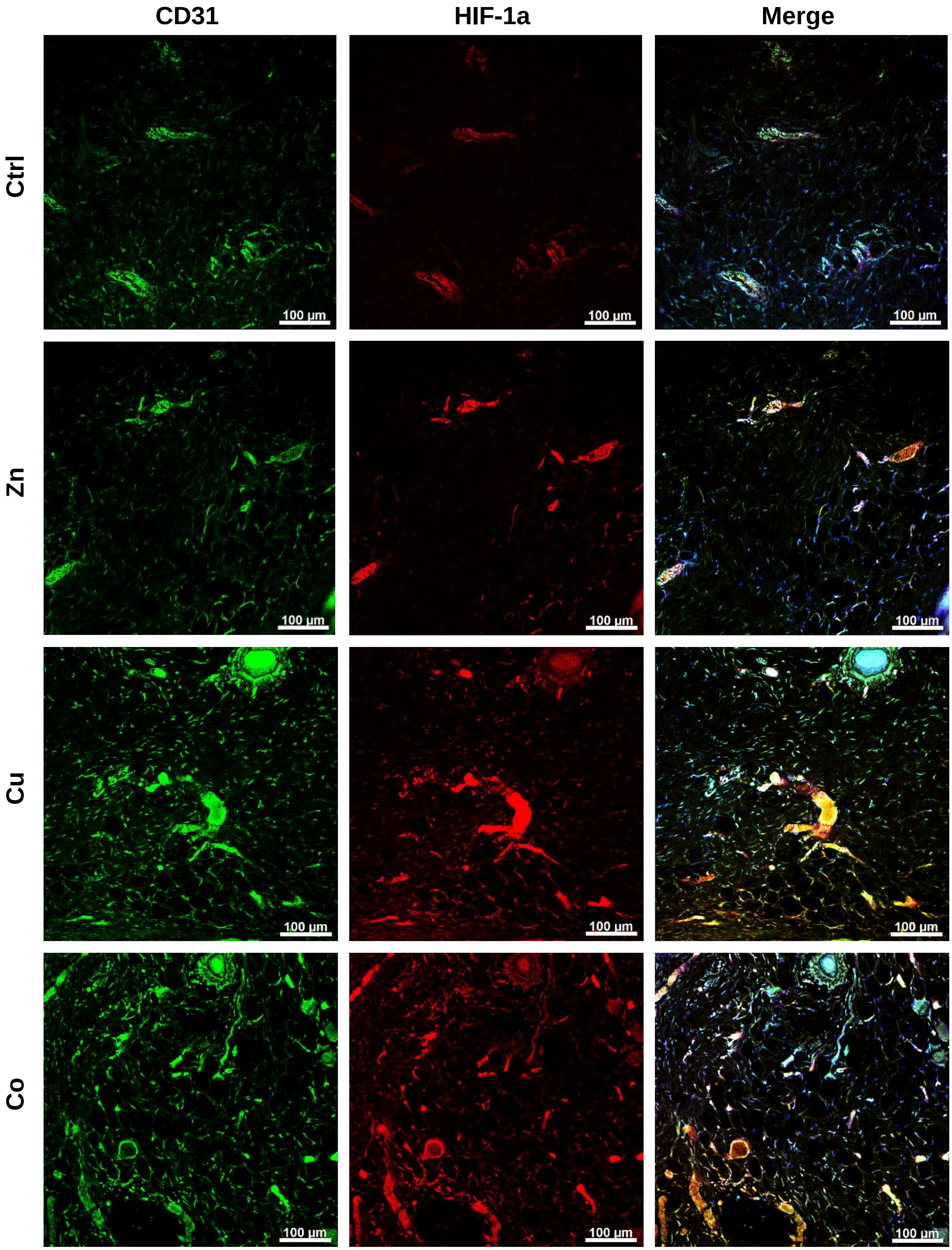

Figure S5. Immunofluorescent analysis of cross-sections of skin explants contacted with subcutaneously implanted TM-doped cryogels at day 5 (Zn and Cu) and day 10 (Ctrl and Co). Simultaneous CD31 CruzFluor™ 488 (green), HIF-1α Alexafluor 647 (red) and DAPI staining was performed.

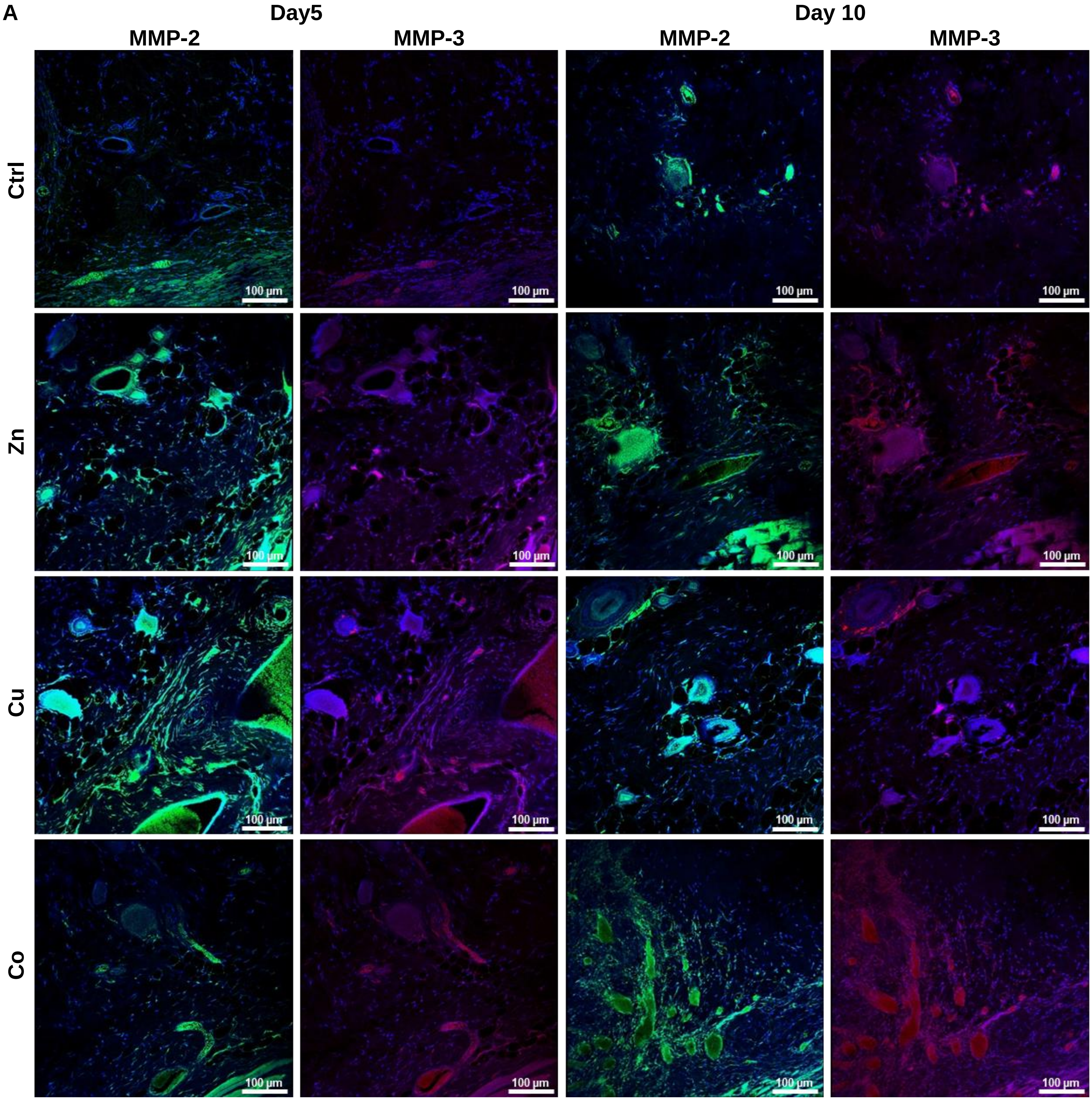
A
Day5
Day 10
MMP-2
MMP-3
MMP-2
MMP-3
Ctrl
Zn
Cu
Co
100 µm

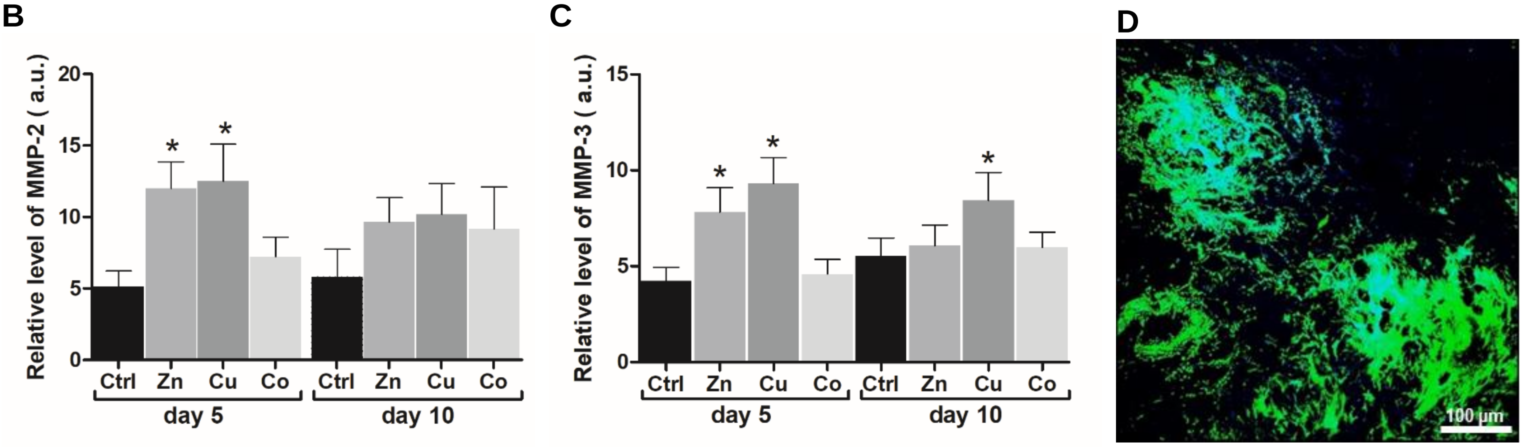

Figure S6. A. Immunofluorescent analysis of cross-sections of skin explants contacted with subcutaneously implanted TM-doped cryogels (MMP-2 AlexaFluor 488 (green) and MMP-3 Alexafluor 647 (red)). B, C – Relative MMP levels in the dermis per field of view (mean ± SD, *$p < 0.05$). D. The area with localized giant cells (MMP-2, Co-doped cryogel).

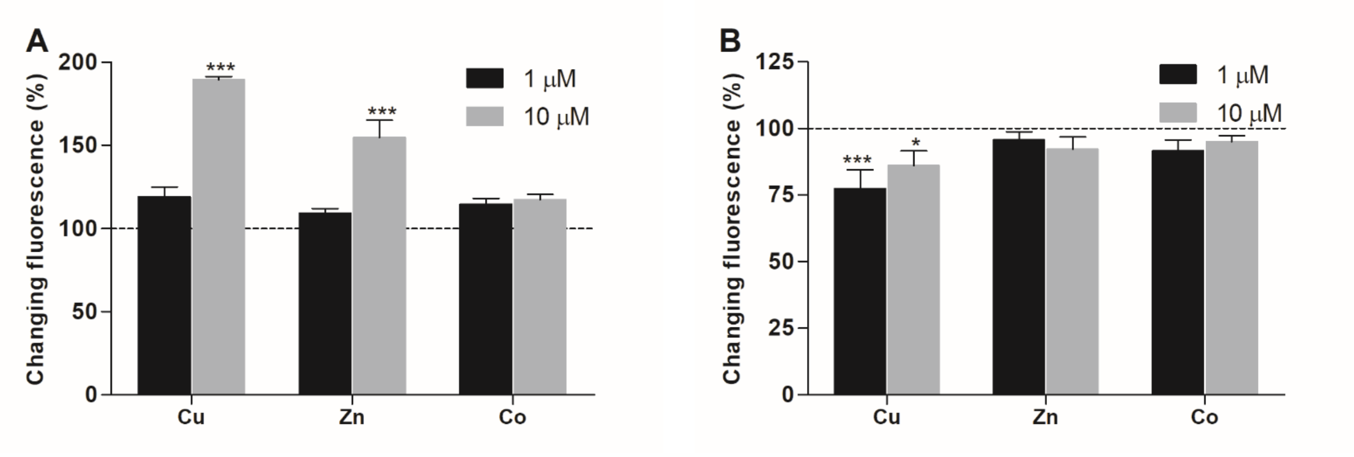

Figure S7. Effect of dissolved metals on relative levels of (A) ROS and (B) reduced glutathione in 3T3 fibroblasts according to DCFDA ($\lambda_{ex}/\lambda_{em}$ = 490/526) and monochlorobimane ($\lambda_{ex}/\lambda_{em}$ = 380/480) fluorescence, respectively. The cells were exposed to metal compounds for 1 h in HBSS. The values were presented as a relative fluorescence signal against the control cells with 100% fluorescence (mean ± SD, $n = 3$, *$p < 0.05$, ***$p < 0.001$).
